# The activity of HYDROPEROXIDE LYASE 1 regulates accumulation of galactolipids containing 12-oxo-phytodienoic acid in Arabidopsis

**DOI:** 10.1093/jxb/erw278

**Published:** 2016-07-15

**Authors:** Anders K. Nilsson, Per Fahlberg, Oskar N. Johansson, Mats Hamberg, Mats X. Andersson, Mats Ellerström

**Affiliations:** ^1^Department of Biological and Environmental Sciences, University of Gothenburg, Box 461, SE-405 30 Göteborg, Sweden; ^2^Division of Chemistry II, Department of Medical Biochemistry and Biophysics, Karolinska Institutet, SE-17 177 Stockholm, Sweden

**Keywords:** Acyl-MGDG, arabidopsides, Arabidopsis accessions, hydroperoxide lyase, jasmonates, OPDA, natural variation.

## Abstract

Arabidopsis produces galactolipids containing esters of 12-oxo-phytodienoic acid (OPDA) and dinor-12-oxo-phytodienoic acid (dnOPDA). These lipids are referred to as arabidopsides and accumulate in response to abiotic and biotic stress. We explored the natural genetic variation found in 14 different Arabidopsis accessions to identify genes involved in the formation of arabidopsides. The accession C24 was identified as a poor accumulator of arabidopsides whereas the commonly used accession Col-0 was found to accumulate comparably large amounts of arabidopsides in response to tissue damage. A quantitative trait loci analysis of an F_2_ population created from a cross between C24 and Col-0 located a region on chromosome four strongly linked to the capacity to form arabidopsides. Expression analysis of *HYDROPEROXIDE LYASE 1* (*HPL1*) showed large differences in transcript abundance between accessions. Transformation of Col-0 plants with the C24 *HPL1* allele under transcriptional regulation of the 35S promoter revealed a strong negative correlation between *HPL1* expression and arabidopside accumulation after tissue damage, thereby strengthening the view that HPL1 competes with ALLENE OXIDE SYNTHASE (AOS) for lipid-bound hydroperoxide fatty acids. We further show that the last step in the synthesis of galactolipid-bound OPDA and dnOPDA from unstable allene oxides is exclusively enzyme-catalyzed and not the result of spontaneous cyclization. Thus, the results presented here together with previous studies suggest that all steps in arabidopside biosynthesis are enzyme-dependent and apparently all reactions can take place with substrates being esterified to galactolipids.

## Highlight

The ability to accumulate OPDA-containing galactoplids in response to wounding was investigated in Arabidopsis accessions and found to correlate with the expression level of the *HYDROPEROXIDE LYASE 1* gene.

## Introduction

Enzymatic or non-enzymatic peroxidation of polyunsaturated fatty acids give rise to a group of products collectively known as oxylipins (Mosblech *et al.*, 2009). The jasmonates are a group of plant-specific oxylipins that have been extensively studied for their involvement in various developmental processes and stress responses (Wasternack and Hause, 2013). The best characterized jasmonates include the phytohormone jasmonic acid (JA), its derivatives methyl jasmonate (MeJA) and jasmonoyl-isoleucine (JA-Ile), and their precursors 12-oxo-phytodienoic acid (OPDA, 18C) and dinor-12-oxo-phytodienoic acid (dnOPDA, 16C) (Acosta and Farmer, 2010). Jasmonate synthesis is initiated in the plastid by hydrolytic liberation of α-linolenic acid (18:3, number of carbons atoms: number of double bonds) or hexadecatrienoic acid (16:3) from membrane lipids like mono- and digalactosyldiacylglycerol (MGDG and DGDG, respectively). 13-lipoxygenase (13-LOX) introduces molecular oxygen to the 18:3/16:3 fatty acid chain and produces 13(*S*)-hydroperoxy-octadecatrienoic acid (13-HPOT)/11(*S*)-hydroperoxy-hexadecatrienoic acid (11-HPHT), which is subsequently converted to 12, 13(*S*)-epoxy-octadecatrienoic acid (12, 13-EOT)/10, 11(*S*)-epoxy-hexadecatrienoic acid (10, 11-EHT) by an allene oxide synthase (AOS). The allene oxide can either undergo spontaneous cyclization into OPDA/dnOPDA, or be enzymatically cyclized by allene oxide cyclase (AOC) into *ditto* products (Schaller *et al.*, 2008; Schaller and Stintzi, 2009; Gfeller *et al.*, 2010). Enzymatically driven cyclization of the unstable allene oxide gives rise to optically pure 9S,13S-OPDA whereas spontaneous cyclization generates a mixture of 9R,13R-OPDA and 9S,13S-OPDA enantiomers (Hofmann *et al.*, 2006). OPDA and dnOPDA [from hereon collectively referred to as (dn)OPDA] are recognized as the first compounds of the synthesis pathway that possess signaling properties in plants (Stintzi *et al.*, 2001; Taki *et al.*, 2005; Goetz *et al.*, 2012; Park *et al.*, 2013; Stotz *et al.*, 2013). To complete synthesis of JA, (dn)OPDA is transported out of the plastid and into the peroxisome where it is reduced and undergoes chain shortening through β-oxidation (Gfeller *et al.*, 2010). Finally, JA is activated by conjugation to amino acids of which L-isoleucine is of particular importance (Staswick and Tiryaki, 2004; Staswick, 2008).

An alternative fate for the lipoxygenase-derived (dn)HPOTs in most plants, including *Arabidopsis thaliana* (from hereon Arabidopsis), is cleavage by hydroperoxide lyase (HPL) into short-chain aldehydes. The products of 13-HPOT are a C_6_ aldehyde [(*Z*)-3-hexenal] and a C_12_ oxo-fatty acid [12-oxo-(*Z*)-9-dodecenoic acid, a.k.a. (9*Z*)-traumatin] whereas dn-13-HPOT forms 10-oxo-(*Z*)-7-decenoic, a.k.a. (7*Z*)-dinortraumatin (Nakashima *et al.*, 2013). The C_6_ aldehydes can be further metabolized to a cocktail of so-called green leaf volatiles, which have well established roles in wounding and defense responses in plants (Matsui, 2006). In addition to the AOS and the HPL branches of the LOX pathway, several other hydroperoxide-metabolizing enzymes have been recognized, including divinyl ether synthase (DES), peroxygenases and epoxy alcohol synthase (Feussner and Wasternack, 2002). Taken together, these pathways give rise to a complex blend of oxylipins.

In Arabidopsis and certain other plant species, mainly from the Brassicaceae family, (dn)OPDA can be found as esters on the glycerol backbone and/or acylated to the 6′-hydroxyl group of the sugar moiety of galactolipids (Stelmach *et al.*, 2001; Hisamatsu et al., 2003, 2005; Andersson *et al.*, 2006; Buseman *et al.*, 2006; Nakajyo *et al.*, 2006; Böttcher and Weiler, 2007; Kourtchenko *et al.*, 2007; Nilsson *et al.*, 2015). These lipids are sometimes referred to as arabidopsides. Synthesis of (dn)OPDA-containing lipids is completed while the fatty acids remain bound to the glycerol and does not require a free fatty intermediate (Nilsson *et al.*, 2012). Moreover, arabidopside synthesis specifically depends on the LOX2 isoform of the 13-LOX for oxidation of the bound fatty acids (Glauser *et al.*, 2009). Not only (dn)OPDA-containing MGDG but also non-oxidized MGDG can be subjected to fatty acylation of the galactose head group, yielding acyl-MGDG. The existence of acyl-MGDG in plants has been known for nearly half a century (Heinz, 1967; Heinz and Tulloch, 1969), and while the prevalence of arabidopsides is rare in the plant kingdom, most, if not all, plant species appear to be able to produce acyl-MGDG (Nilsson *et al.*, 2015). Several types of biotic and abiotic stresses induce the formation of arabidopsides and acyl-MGDG in Arabidopsis, including pathogen elicitation (Andersson *et al.*, 2006; Kourtchenko *et al.*, 2007; Zoeller *et al.*, 2012; Vu *et al.*, 2012; Nilsson *et al.*, 2014), mechanical wounding (Stelmach *et al.*, 2001; Buseman *et al.*, 2006; Böttcher and Weiler, 2007; Ibrahim *et al.*, 2011; Nilsson *et al.*, 2012) and low temperatures (Vu *et al.*, 2012, 2014). No clear biological role has yet been ascribed to the formation of acyl-MGDG in plants. Arabidopsides, on the other hand, possess antimicrobial activity *in vitro* (Andersson *et al.*, 2006; Kourtchenko *et al.*, 2007) and have been suggested to play a role in plant herbivore perception through the release of free OPDA during insect feeding (Schafer *et al.*, 2011). Furthermore, two lipases in Arabidopsis, PLAI and pPLAIIα, have been found to utilize arabidopsides as substrates and catalyze the release of free OPDA (Yang *et al.*, 2007*b*, 2012). The mechanism by which arabidopside production is activated and how the synthesis is regulated however remains obscure.

Arabidopsis is found in nature throughout the Eurasian continent in diverse habitats. This has given rise to an abundance of ecotypes, also called accessions, which vary considerably in form, development and physiology. Natural variation in plants has been extensively used as a means to identify genes that underlie complex traits (e.g. Mendez-Vigo *et al.*, 2013). Experimental genetic work with Arabidopsis accessions has been greatly facilitated by the launch of the 1001 Genomes Project and the release of a great number of whole-genome sequences (http://1001genomes.org/; Weigel and Mott, 2009; Cao *et al.*, 2011; Ledford, 2011).

In the present study, we explore natural variation among Arabidopsis accessions with respect to arabidopsides and acyl-MGDG. We found that the ability to accumulate these lipids in response to tissue disruption is highly variable between accessions and we propose that *HPL1* plays a major role in regulating the levels of arabidopsides.

## Material and methods

### Plant material and growth conditions

Arabidopsis seeds were sown on soil and stratified for 2–4 d at 4 °C to break dormancy. The plants were cultivated in controlled growth chambers under an 8h photoperiod, 22/18 °C day/night temperature and 60% relative humidity. The Arabidopsis accessions were a kind gift from Prof. Mary Beth Mudgett (Stanford University). Arabidopsis *Agrobacterium*-transferred DNA (T-DNA) insertion lines were acquired from the Nottingham Arabidopsis Stock Centre (Scholl *et al.*, 2000; Alonso *et al.*, 2003) and tested to be homozygous using primers designed from T-DNA Express (http://signal.salk.edu/cgi-bin/tdnaexpress). The *lox2-1* loss-of-function mutant (Glauser *et al.*, 2009) was a generous gift from Prof. Edward Farmer (University of Lausanne). Col-0 *lox2-1* was crossed to C24 and homozygous *lox2-1* plants in the F_2_ generation were identified using allele specific digestion of PCR amplified gene fragments (Glauser *et al.*, 2009).

### Extraction and quantification of lipids

A total lipid extract from 2–4 leaf discs with a 7mm diameter was isolated as described (Kourtchenko *et al.*, 2007) and dissolved in 50 µl methanol. The lipids were analyzed using an Agilent 1260 HPLC system coupled to an Agilent 6410 triple quadrupole detector equipped with an electrospray interface as previously described (Nilsson *et al.*, 2014, 2015). Neutral loss of headgroup specific fragments of the ammoniated adducts of each lipid was used for detection (Ibrahim *et al.*, 2011) (Supplementary Table S1 at *JXB* online). Quantification was achieved using external standard curves constructed from purified arabidopside A, B and E (Andersson *et al.*, 2006; Kourtchenko *et al.*, 2007), tri-18:3 acylated MGDG (Nilsson *et al.*, 2015) and commercially available spinach leaf MGDG and DGDG (Larodan, Malmö, Sweden). Each analyzed lipid species was quantified against the calibration curve for the most structurally related standard as indicated in Supplementary Table S1. Extraction and quantification of arabidopsides in the mapping population were as previously described (Kourtchenko *et al.*, 2007). Total esterified OPDA was extracted, transmethylated and quantified as previously described but using a total lipid extract (Nilsson *et al.*, 2012).

### Steric analysis

A total lipid extract from 1g Arabidopsis leaf tissue was prepared as described (Kourtchenko *et al.*, 2007) and the galactolipid fraction was isolated by silicic acid column chromatography (elution with acetone). Material obtained following treatment with 0.5M NaOH in 75% ethanol at 50 °C for 30min was treated with diazomethane and analyzed by GC-MS. Four peaks of oxylipins were observed, i.e. the methyl esters of 13-iso-OPDA, 13-iso-dnOPDA, Δ^9(13)^-OPDA, and Δ^9(13)^-dnOPDA (cf. Andersson *et al.*, 2006). The two first-mentioned compounds were isolated in pure form by SP-HPLC using 0.75% 2-propanol-hexane as mobile phase and subsequently analyzed by CP-HPLC using a Chiralcel OB-H column (250×4.6mm) and 3% 2-propanol (13-iso-OPDA) or 10% 2-propanol (13-iso-dnOPDA) in hexane for the elution. Detection was at 220nm.

### Quantitative real-time PCR

RNA from leaf tissue of 8-week-old plants was extracted using the RNeasy Plant Mini Kit (Qiagen, Germany). RNA concentration and quality were determined with NanoDrop (Thermo scientific, USA) and 2200 TapeStation (Agilent, USA) respectively. Samples were stored at −80 °C before further use. 2 µg of total RNA were subjected to DNase treatment (DNase I, Life technologies, USA) and cDNA synthesis using oligo(dT)18 primers with Superscript III Reverse Transcriptase (Life technologies, USA). 1–2 µl of 2× diluted cDNA and primers were mixed with iTaq Fast SYBR Green Supermix with ROX (Bio-Rad, USA) in 15 µl reactions according to the manufacturer’s instructions. Quantitative real-time PCR (qPCR) analysis was performed on a C1000 Touch Thermal Cycler instrument (Bio-Rad, USA). Amplifications were performed in two-step PCR with the conditions 95 °C for 3min followed by 40 cycles of 95 °C for 5s and 60 °C for 30s. Melt-curve analyses were performed for all primers after amplification and expected product sizes were confirmed by agarose gel electrophoresis. PCR amplification efficiencies of all target genes were established from serial diluted calibration curves of Col-0 or C24 cDNA. Primers used for the quantification of the reference gene *PEROXIN 4* (*PEX4*, At5G25760) and *LOX2* (At3G45140) were those described by Glauser *et al.*, (2009). *PEX4* was found to be similarly expressed in Col-0, C24 and L*er* and was therefore used as reference gene in all experiments. Primers for *AOS* (At5G42650), *AOC1* (At3G25760), *AOC2* (At3G25770), *AOC3* (At3G25780) and *AOC4* (At1G13280) are presented in Supplementary Table S2. All primers were controlled not to bind any SNP containing regions in C24 or L*er*. Efficiencies from calibration curves were determined using BioRad CFX manager 3.0 software (Bio-Rad, USA). Relative expression was calculated by subtracting the average Cq value of the reference gene based on two technical and three biological replicates from the Cq value of target gene (ΔCq). Fold change was calculated as 2^− ΔCq^. Difference in normalized gene expression was statistically analyzed using one-way ANOVA with Tukey’s post hoc test with *P*<0.05 considered significant.

### Genetic mapping

Col-0 was crossed to C24 and the F_1_ population was left to self-pollinate. Galactolipids were extracted from ~200 individual (7–8-week-old) F_2_ plants 60min after freeze-thaw wounding as previously described (Kourtchenko *et al.*, 2007; Nilsson *et al.*, 2012). Arabidopside A, B, E and G were quantified (Kourtchenko *et al.*, 2007), summarized and the data was used for the quantitative trait loci (QTL) analysis. PCR marker primers were designed using Primer3 (http://primer3.wi.mit.edu/) from available Col-0 and C24 sequence data (http://www.TAIR.org/, http://www.1001genomes.org/). Three types of genetic PCR markers were used for the QTL analysis and the fine-mapping: simple sequence length polymorphisms (SSLP), allele specific single-nucleotide polymorphism and tetra-primer amplification refractory mutation system (TP-ARMS) (Ye *et al.* 2001). PCRs were performed with a MyCycler and a S1000 Thermal Cycler (both Bio-Rad, USA) using Titanium *Taq* polymerase (Clontech, USA) with one quarter of the amount of enzyme recommended by the manufacturer. PCR products were analyzed by agarose gel electrophoresis (2–4% SeaKem LE Agarose depending on the product size). A complete list of marker names, genetic positions and primer sequences are presented in Supplementary Table S3. The QTL analysis was performed using the software QTLNetwork 2.0 (Yang *et al.*, 2007*a*, 2008). When interesting crossing-over events were observed but the phenotype was ambiguous in F_2_ plants, the plants were left to self-pollinate and the segregation pattern in terms of arabidopside accumulation was monitored in the subsequent F_3_ population.

### Cloning of C24 *HPL1*

The C24 *HPL1* (AT4G15440) allele was PCR amplified from cDNA using AccuPrime *Pfx* DNA polymerase (Life technologies, USA) with forward primer GA*ACTAGT*ATGTTGTTGAGAACGATGGCG and reverse primer GA*GGGCCC*TTATTTAGCTTTAACAACAGCTTT (italic letters indicate SpeI and ApaI restriction enzyme cleavage sites). The PCR product was blunt-end cloned into vector pJET1.3 (Thermo Fisher Scientific), digested using restriction enzymes SpeI and ApaI (New England Biolabs, USA), agarose gel purified, and ligated (T4 DNA ligase, Life technologies, USA) into the vector pB2GW7 (Karimi *et al.*, 2002) linearized with the same enzymes. The full length clone was verified by sequencing (Eurofins MWG Operon, Germany) using available C24 sequence scaffolds (http://www.1001genomes.org) and TAIR10 data (http://www.arabidopsis.org/) (Supplementary Fig. S1). The *HPL*-containing vector and empty vector pB2GW7 were electroporated into *Agrobacterium tumefaciens* strain GV3101 and subsequently transformed to Col-0 plants using the floral dip method (Clough and Bent, 1998). Successfully transformed plants of the first generation were selected by spraying seedlings at the four leaf stage with 120mg l^−1^ glufosinate (Basta, AgrEvo, Germany) with 0.02% Tween 20.

## Results

### Natural variation among Arabidopsis accessions in OPDA-containing galactolipid formation

**Fig. 1.**
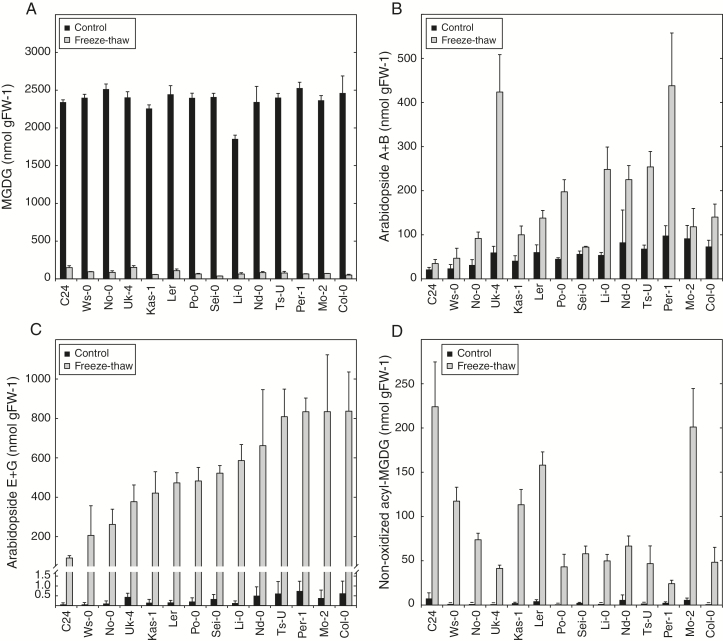
Natural variation in wounding-induced galactolipids. Leaf discs from 14 different Arabidopsis accessions cultivated for 8 weeks were frozen in liquid nitrogen (control) and left to thaw at room temperature for 60min (freeze-thaw). The lipids were extracted and quantified with LC-MS/MS. (A) Sum of the most abundant MGDG species (34:3, 34:4, 34:5, 34:6, 36:4, 36:5 and 36:6). (B) Sum of two most abundant OPDA-containing MGDG species arabidopside A (OPDA, dnOPDA) and arabidopside B (OPDA, OPDA). (C) Sum of the two most abundant acylated OPDA-containing MGDG species arabidopside E (OPDA, dnOPDA, OPDA) and arabidopside G (OPDA, OPDA, OPDA). (D) Sum of the most abundant non-oxidized acylated MGDG species (50:6, 50:9, 52:9 and 54:9). The average and standard deviation of triplicate samples are shown.

We set out to investigate how the galactolipid profile changes in response to wounding in naturally occurring populations of Arabidopsis. Fourteen different accessions collected from geographically dispersed locations were selected for the analysis. Leaf tissue from 8-week-old plants was snap-frozen in liquid nitrogen and analyzed for galactolipids by LC-MS/MS. In parallel, samples were left to thaw for 1h at room temperature before lipid extraction, a procedure which causes loss of cellular integrity and triggers a strong wounding response and accumulation of (dn)OPDA containing galactolipids (Fig. 1; Supplementary Table S1) (Nilsson *et al.*, 2012). All accessions contained comparable levels of MGDG before wounding (Fig. 1A). Between 93 and 98% of the MGDG was lost irrespective of genetic background after the freeze-thaw treatment. The trienoic fatty acids 18:3 and 16:3 of the MGDG were partly converted into OPDA and dnOPDA in all accessions. However, levels of arabidopsides of types A and B (no acylation on the galactose moiety) and types E and G (acylated with OPDA on the galactose moiety) varied significantly among accessions (Fig. 1B and C, respectively). Col-0 was found to accumulate almost 10-fold more arabidopside E and G compared to C24. Interestingly, C24 accumulated more non-oxidized acyl-MGDG after tissue disruption than any of the other tested accessions (Fig. 1D). Overall, C24 and Col-0 displayed the largest difference in galactolipid signatures and were therefore selected for further studies.

### LOX2-dependent and -independent metabolism of galactolipid-bound fatty acids after tissue disruption

**Fig. 2.**
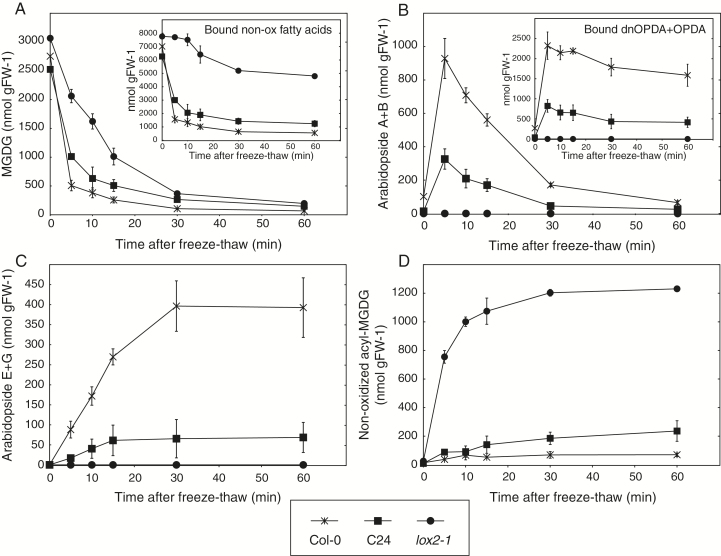
Breakdown of MGDG in Col-0, C24 and *lox2-1* after tissue disruption. Leaf discs obtained from 8-week-old Arabidopsis were frozen (control) and thawed for indicated time points (freeze-thaw) and then lipids were extracted and quantified by LC-MS/MS. Shown is (A) the sum of the most abundant MGDG species; (B) arabidopside A and B; (C) arabidopside E and G; (D) non-oxidized acyl-MGDG species. The concentrations of galactolipid bound non-oxidized fatty acids and (dn)OPDA were calculated from lipid species presented in Supplementary Table S1 (inserts of A and B, respectively). The average and standard deviation of triplicate samples are shown.

In order to study the kinetics of wounding-induced lipid metabolism in closer detail, leaf tissue from C24 and Col-0 was frozen in liquid nitrogen and then thawed for different periods of time prior to lipid extraction. The *lox2-1* mutant in the Col-0 genetic background, unable to synthesize lipid-bound (dn)OPDA (Glauser *et al.*, 2009), was also included in the experiment. Approximately one third of the MGDG was converted into other compounds in *lox2-1* samples within 5min after wounding (Fig. 2A). The loss of MGDG did not correlate with fatty acid oxidation (Fig. 2A, insert) but instead to a rapid accumulation of non-oxidized acyl-MGDG (Fig. 2D). In comparison, only 40% and 20% of the original tissue concentration of MGDG was left after 5min of thawing in C24 and Col-0, respectively. Levels of arabidopsides peaked within the first few minutes and then slowly declined in both Col-0 and C24 (Fig. 2B, and insert). Levels of arabidopsides E and G increased at the expense of arabidopsides A and B in both accessions during the first 30min (Fig. 2B, C). This result is in accordance with our previous report on galactolipid-bound OPDA (Nilsson *et al.*, 2012).

### LOX2 determines MGDG degradation in both Col-0 and C24 in response to wounding

**Fig. 3.**
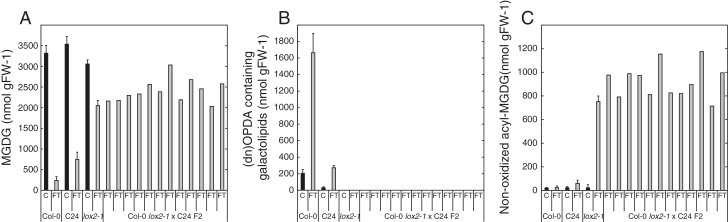
*LOX2*-dependent breakdown of MGDG. Leaf discs obtained from 8-week-old Arabidopsis were frozen (C, control) and thawed for 5min (FT, freeze-thaw) and then lipids were extracted and quantified by LC-MS/MS. Shown is (A) the sum of the most abundant MGDG species; (B) all detected (dn)OPDA-containing galactolipid species; (C) non-oxidized acyl-MGDG species. The average and standard deviation of triplicate samples are shown for Col-0, C24 and *lox2-1*. Concentrations of single samples are shown for Col *lox2*-1 × C24 F_2_ plants.

Approximately 6 µmol g FW^−1^ of the galactolipid-bound fatty acids were subjected to enzymatic peroxidation by LOX2, or metabolized through factors regulated by LOX2 during the first few minutes following wounding in wild-type Col-0 (Fig. 2A, insert). This finding encouraged us to test whether LOX2 regulates breakdown of MGDG also in the C24 accession. The *lox2-1* mutant in Col-0 background was crossed to C24 and the F_1_ plants were allowed to self-pollinate. Twelve plants homozygous for the *lox2-1* mutation from the F_2_ population were examined for galactolipids 5min after freeze-thaw (Fig. 3). All of the F_2_ plants metabolized MGDG to the same extent as *lox2-1* (Fig. 3A). Moreover, the F_2_ plants were inseparable from *lox2-1* plants in terms of arabidopsides and acyl-MGDG (Fig. 3B, C). Taken together, these results strongly indicate that the *LOX2* gene regulates MGDG breakdown similarly in Col-0 and C24 in response to loss of cellular integrity.

### Galactolipid-bound OPDA is exclusively made through enzymatic cyclization of the precursor epoxy acid

**Fig. 4.**
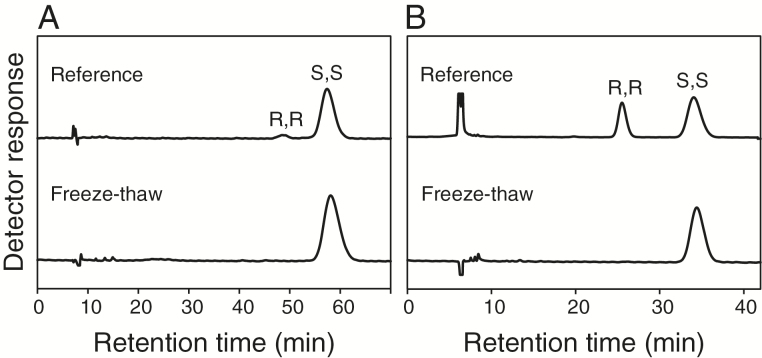
Steric analysis of OPDA and dnOPDA after freeze-thaw wounding. One gram of Arabidopsis leaf tissue was snap-frozen in liquid nitrogen and left to thaw for 1h at room temperature. Galactolipids were extracted, hydrolyzed and methyl-esterified, and the methyl esters of 13-iso-OPDA and 13-iso-dnOPDA were isolated by SP-HPLC. Aliquots of these materials were subjected to chiral-phase HPLC. The methyl esters of (A) 13-iso-OPDA and (B) 13-iso-dnOPDA are shown. The top curve in each graph shows a reference sample prepared from OPDA or dnOPDA containing both the natural 9S,13S form and the 9R,13R form.

AOS is encoded by a single locus that is strictly necessary for the synthesis of arabidopsides and other jasmonates (Laudert *et al.*, 1996; Nilsson *et al.*, 2012). Arabidopsis contains four genes that codes for AOC enzymes (*AOC1-4*) (Stenzel *et al.*, 2003). All four AOC members can, together with AOS, synthesize OPDA from free or methylated HPOT at similar rates *in vitro* (Schaller *et al.*, 2008). Their ability to use glycerolipid-bound HPOT as substrate has not been reported. We investigated the chirality of lipid-bound OPDA and dnOPDA after tissue damage to establish whether or not they are formed through enzymatic cyclization of 12, 13-EOT/10, 11-EHT. Leaf tissue from Col-0 was snap-frozen and left to thaw for 60min whereafter the galactolipid fraction was extracted and hydrolyzed. Fatty acids were subsequently methylated and separated on chiral-phase HPLC (Fig. 4). Both OPDA (Fig. 4A) and dnOPDA (Fig. 4B) were found to be stereochemically pure and to occur in the natural 9S,13S form. Thus, arabidopsides are apparently formed exclusively from enzymatic cyclization by AOC while the fatty acid remains attached to the glycerol backbone.

### No difference in expression of (dn)OPDA biosynthesis genes between accessions can be observed using qPCR

**Fig. 5.**
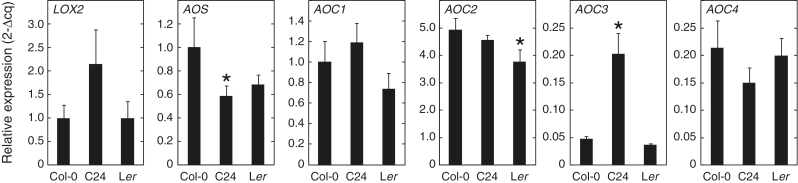
qPCR of jasmonate biosynthesis genes. Total RNA from 8-week-old Arabidopsis plants was extracted and subjected to cDNA synthesis. The relative expression of *LIPOXYGENASE 2* (*LOX2*), *ALLENE OXIDE SYNTHASE* (*AOS*) and *ALLENE OXIDE CYCLASES 1*, *2*, *3* and *4* (*AOC1*, *AOC2*, *AOC3* and *AOC4*) was determined by qPCR. Expression was compared to that of the reference gene *PEROXIN 4* (*PEX4*, At5G25760). Relative expression levels for *LOX2* and *AOS* were normalized to that of Col-0 for each respective gene. Relative expression levels for *AOC1*, *AOC2*, *AOC3* and *AOC4* were normalized to that of *AOC1* in Col-0. Averages based on three biological replicates are shown. Error bars represent standard deviation. Asterisk denotes *P*<0.05 statistically significant difference from Col-0 as determined by one-way ANOVA with Tukey’s post hoc test.

The extremely fast production of arabidopsides in response to wounding suggests that a preformed biosynthetic machinery exists, and that this machinery is activated upon tissue disruption. Thus, we hypothesized that differential expression of (dn)OPDA biosynthesis genes during basal conditions could explain the variation in arabidopside accumulation between accessions; high expression of genes regulating (dn)OPDA synthesis would result in increased arabidopside levels. To test this, qPCR was performed for *LOX2*, *AOS* and the four isoforms of *AOC*, in the accessions Col-0, C24 and L*er*. These accessions were selected based on the finding that the freeze-thaw treatment caused Col-0 plants to accumulate 5–10-fold more galactolipid-bound (dn)OPDA than C24, whereas the L*er* accession produced intermediate amounts of arabidopsides compared to Col-0 and C24 (Fig. 1). Primers used in the experiment were designed to not span any sequence polymorphisms that existed between accessions. The relative expression of *LOX2* and *AOS* were normalized to Col-0 transcription of the respective gene. *AOC2*, *AOC3* and *AOC4* were normalized to the Col-0 expression of *AOC1*. The expression profiles of all six genes were overall very similar for the three accessions (Fig. 5), suggesting that regulation of basal transcription of the genes encoding (dn)OPDA-synthesis enzymes plays a minor role in determining different accessions’ ability to accumulate arabidopsides.

### Genetic mapping of genes influencing arabidopside accumulation

**Fig. 6.**
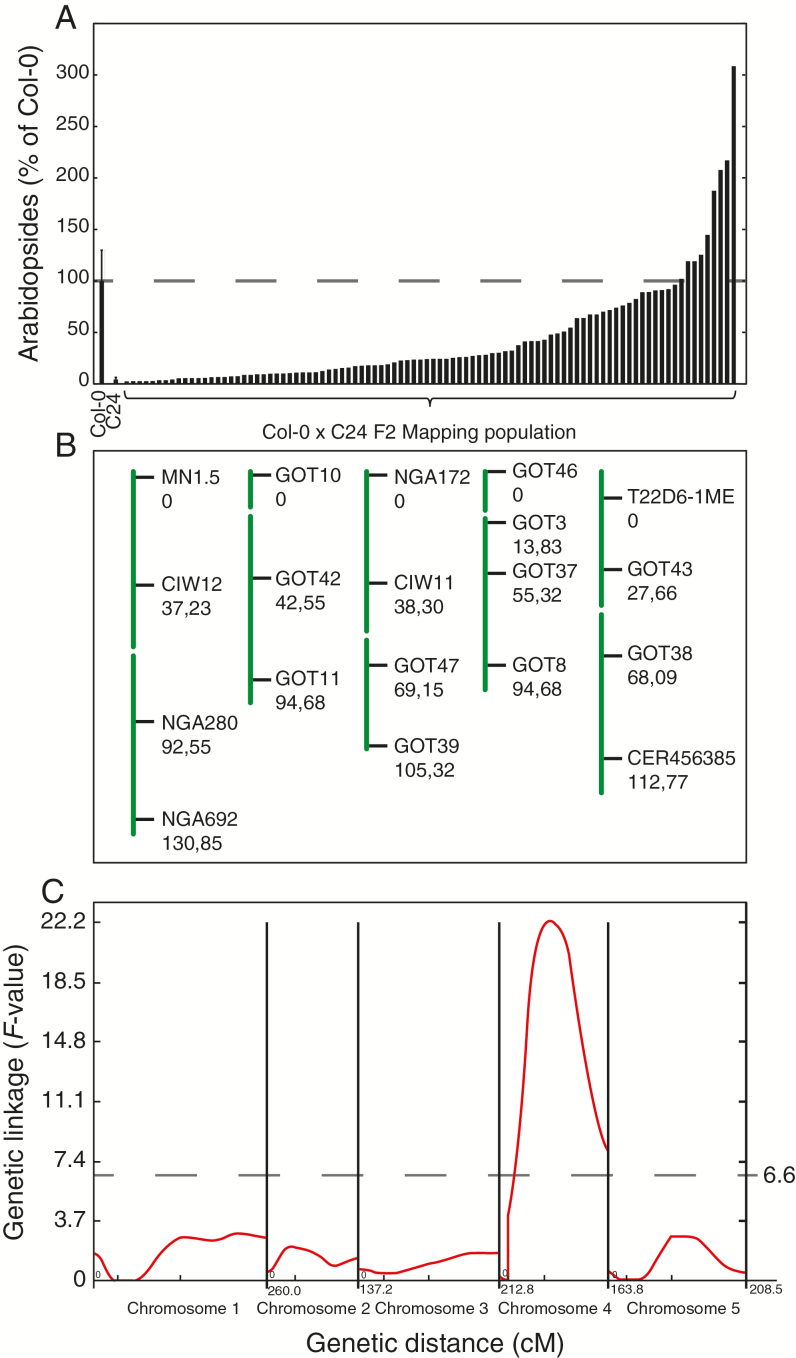
Mapping genes involved in arabidopside synthesis. A quantitative trait loci (QTL) analysis was performed based on genetic and phenotypic data retrieved from 94 individual Col-0 × C24 F_2_ plants. (A) The amount of arabidopside A, B, E and G were determined in 7–8-week-old F_2_ plants 60min after freeze-thaw treatment. Averages and standard deviation (*n*=11) for Col-0 and C24 samples are shown. (B) The plants were genotyped using 19 PCR markers. The genetic distances in centimorgan (cM) based on crossing-over events in the mapping population are shown below the marker names. (C) One QTL with a peak value that exceeded the threshold *F*-value (6.6), calculated by permutation test on chromosome 4, was identified. (This figure is available in color at *JXB* online.)

The lack of transcriptional regulation of genes involved in (dn)OPDA synthesis led us to search for other genetic factors that influence the plants’ ability to synthesize or accumulate arabidopsides. To this end, Col-0 was crossed to C24 to create a mapping population. Approximately 200 individual F_2_ plants were investigated for their ability to produce arabidopsides A, B, E and G 60min after freeze-thaw induced wounding. The levels of arabidopsides in 94 representative plants from this mapping population are shown in Fig. 6A. To identify loci responsible for the large variation in arabidopside accumulation among these 94 F_2_ plants, a PCR-based quantitative trait loci (QTL) analysis was performed. Simple sequence length polymorphisms (SSLPs) markers evenly distributed across the Arabidopsis genome were used for the QTL analysis (Fig. 6B and Supplementary Table S3). Only one QTL was identified with a peak *F*-value exceeding the calculated significance threshold (>0.05 significance level) (Fig. 6C) (Yang *et al.*, 2007*a*, 2008). The QTL was estimated to be positioned between the two genetic markers GOT3 and GOT8, close to GOT37 on chromosome 4. In order to locate the QTL more accurately, PCR-guided fine-mapping was initiated using SSLP and allele specific SNP markers. The QTL could be confined to a 67 kbp region between the markers GOT35 and GOT6 containing only 21 genes (Supplementary Fig. S2A). Eighteen of the genes in the mapped region were found to have one or more single nucleotide polymorphism (SNP) in C24. Furthermore, 15 of these 18 genes carried SNPs that coded for non-synonymous amino acid substitution. Of the corresponding protein products of the 21 genes, five have been experimentally localized to the chloroplast or predicted to contain a chloroplast transit peptide (Emanuelsson *et al.*, 1999; Lamesch *et al.*, 2012). Col-0 T-DNA insertion mutation lines for 11 of the genes with SNPs that coded for non-synonymous amino acid substitution were tested for arabidopside content in leaf tissue 1h after freeze-thaw treatment (Supplementary Fig. S2B). None of the tested lines were found to have significantly lower levels of arabidopsides than wild type Col-0. One of the genes found in the mapped region with confirmed chloroplast localization was *HPL1*. Col-0 naturally contains a deletion in the first exon of *HPL1* that results in a premature stop codon and a truncated protein product (Duan *et al.*, 2005).

### C24 and L*er* display large difference in *HPL1* expression

**Fig. 7.**
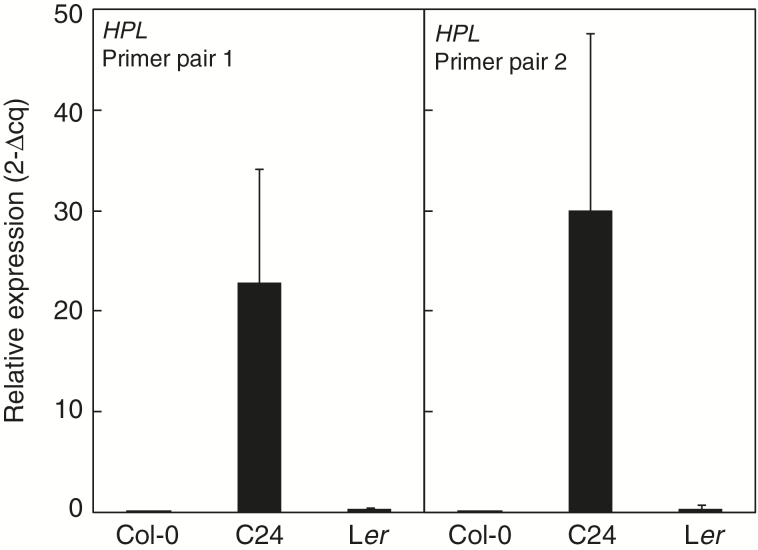
*HPL1* expression in Col-0, C24 and L*er*. Relative expression of *HPL1* to the reference gene *PEX4* was determined by qPCR in Col-0, C24 and L*er*. Two different primers pairs were used for the qPCR analysis: one pair giving rise to a product spanning the *HPL* intron (left chart), and one pair containing an exon-exon spanning primer (right chart). Averages based on three biological replicates and two technical replicates are shown. Error bars represent standard deviation. Expression analysis of *HPL1* in Col-0 and C24 was repeated in one independent experiment with similar results.

The direct link between 13-LOX products and HPL activity impelled us to examine whether the *HPL1* gene might explain the difference in arabidopside accumulation found between accessions. Neither L*er* nor C24 carry the frame-shift causing deletion found in Col-0 *HPL1*, and the protein product of L*er HPL1* has been shown to be enzymatically active *in planta* (Supplementary Fig. S1; Duan *et al.*, 2005). Although both C24 and L*er* have functional *HPL1* alleles, their ability to accumulate arabidopsides differs substantially. To address this, we determined transcript levels of *HPL1* in Col-0, C24 and L*er* with real-time qPCR using two alternative primer pairs (Fig. 7). The two independent qPCR reactions gave similar results: the C24 *HPL1* allele was expressed ~80-fold higher than the corresponding L*er* allele.

### *HPL1* expression is proportional to accumulation of OPDA-containing galactolipids

**Fig. 8.**
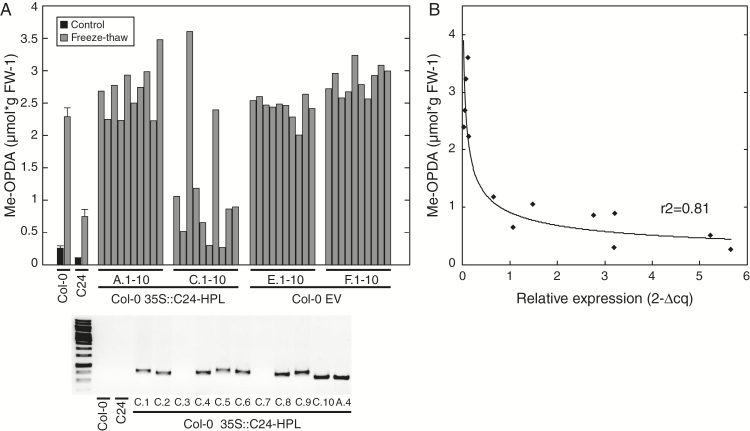
HPL as a regulator of arabidopside accumulation. (A) Leaf discs from Col-0 plants of the T2 generation, transformed with either the C24 *HPL* allele driven by the 35S promoter (Col-0 35S::C24-HPL) or an empty vector (Col-0 EV), were analyzed for glycerolipid bound OPDA 60min after freeze-thaw treatment by GC-MS. Ten individual T2 plants originating from four different T1 plants (of which two are shown in figure) were analyzed for the overexpressing line (Col-0 35S::C24-HPL A and C, respectively). Ten individual T2 plants originating from two different T1 plants were analyzed for the empty vector control (Col-0 EV E and F). Standard deviation and averages based on three replicates are shown for Col-0 and C24. Presence of the 35S::C24-HPL construct was verified by PCR using primers binding the 35S promoter (left primer) and *HPL1* gene (right primer) and the product detected after agarose gel electrophoresis (A, lower panel). (B) The relative expression of *HPL1* to the reference gene *PEX4* was determined by qPCR in a subset of the Col-0 35S::C24-HPL plants and plotted against the level of lipid bound OPDA. A logarithmic regression line was fitted to the data. The *R*-squared value of the regression is shown inside the graph.

To further investigate the relationship between *HPL1* expression and arabidopside accumulation, the C24 *HPL1* was cloned and transformed into Col-0 plants. In order to test if any *cis* regulatory elements of the C24 *HPL1* allele influenced the exceptionally high expression observed, the full genomic sequence of C24 *HPL1*, including 845bp directly upstream of the start codon, was cloned and transformed via *Agrobacterium tumefaciens* to Col-0 plants. However, when transformants from the T_2_ generation were analyzed for *HPL1* expression by qPCR, transcription was only moderately higher compared to Col-0 control plants and no difference in OPDA esters could be detected (not shown). The very high abundance of *HPL1* transcripts in C24 may thus depend on accession specific *trans*-acting elements. For this reason, we next made a construct from cDNA of the C24 *HPL1* and fused it to the strong constitutive CaMV 35S promoter and subsequently transformed it to Col-0 plants. In parallel, Col-0 plants were also transformed with an empty vector used as control. The presence of the *HPL1* construct in plants of the T_1_ generation was identified using glufosinate selection and confirmed by PCR (not shown). To avoid any effect of the glufosinate selection treatment, plants from the T_2_ generation were used for further experiments. Leaf tissue from plants transformed with either the C24 *HPL1* (Col-0 35S::C24-HPL) or the empty vector (Col-0 EV) were analyzed for glycerolipid-bound OPDA 60min after freeze-thaw treatment with GC-MS (Fig. 8A). Of the Col-0 35S::C24-HPL plants, only the progeny from one out of four tested T_1_ plants showed reduced levels of OPDA-esters (35S::C24-HPL pool C in Fig. 8A). These plants were further analyzed for the presence of the HPL construct by PCR (Fig. 8A, lower panel) and *HPL1* expression by qPCR (Fig. 8B). The two plants from the C pool that accumulated high levels of OPDA-esters after the freeze-thaw treatment, plants C.3 and C.7, were found to not carry the C24-*HPL* construct (Fig. 8A, lower panel). A strong logarithmic correlation relationship (*R*^2^=0.81) between the level of OPDA-esters and *HPL1* expression in the Col-0 35S::C24-HPL plants was apparent (Fig. 8B).

## Discussion

Chloroplast membrane lipids containing (dn)OPDA, so-called arabidopsides, are mainly found in plants of the Brassicaceae family (Böttcher *et al.*, 2007). Acyl-MGDG on the other hand appears to be ubiquitous in the plant kingdom (Heinz, 1967; Vu *et al.*, 2014; Nilsson *et al.*, 2015). It remains enigmatic as to why plants produce these lipids to such an extent following pathogen elicitation and mechanical wounding.

### Natural variation of (dn)OPDA-containing and head group acylated galactolipids

We took use of natural variation in Arabidopsis to gain insight into the genetic machinery that underlies production of arabidopsides and acyl-MGDG. The ability to produce arabidopsides in response to freeze-thaw wounding was found to be highly variable between different Arabidopsis accessions (Fig. 1). Certain accessions (e.g. Uk-4) primarily accumulated non-galactose acylated arabidopside species whereas others (e.g. Col-0) primarily accumulated galactose acylated species. Still others (e.g. Per-1) produced high levels of both types of arabidopsides. Differential expression of the chief enzyme responsible for the acyl transfer of fatty acids to head group of galactolipids (Nilsson *et al.*, 2015) could potentially explain these differences found between accessions. The C24 accession was recognized as a poor arabidopside producer but did instead accumulate relatively high levels of non-oxidized acyl-MGDG species after tissue damage. The finding that C24 produces low amounts of arabidopsides is in agreement with a recent publication (Vu *et al.*, 2014).

*lox2-1* mutant plants, impaired in the initial step of arabidopside synthesis, also accumulated high levels of non-oxidized acyl-MGDG (Fig. 2). Likewise, plants impaired in the second enzymatic step of arabidopside synthesis, *aos* mutants, accumulate non-oxidized acyl-MGDG in favor of acyl-arabidopsides (Nilsson *et al.*, 2014). Intriguingly, the formation of acyl-MGDG species in the *lox2-1* background was markedly faster than the production of acyl-arabidopsides in wild-type Col-0 plants (Fig. 2C, D). There are at least two possible explanations for this: (i) the acyl transferase has higher substrate specificity for non-oxidized fatty acids; or perhaps more likely (ii) the higher availability of substrates for the acyl transferase in *lox2-1* results in a more rapid production of acylated galactolipids. Not all MGDG could be accounted for in Col-0 and C24 after freeze-thaw and it is thus possible that additional species of oxidized and acylated lipids, as well as lyso-MGDG, that we did not measure are formed in these plants. The reduction of non-oxidized fatty acids in the galactolipid pool in the *lox2-1* mutant (Fig. 2A) is probably at least partly the result of increased non-enzymatic lipid oxidation and enzymatic oxidation through the 9-LOX pathway (Zoeller *et al.*, 2012).

### Natural variation in *HPL1* expression

We were able to map the difference in arabidopside accumulation between the accessions Col-0 and C24 to a region on chromosome four corresponding to only 21 genes. Out of the genes found within this QTL, 15 contained SNPs that coded for non-synonymous amino acid substitutions (Supplementary Fig. S2). One of the genes in the mapped QTL, *HPL1*, was identified to negatively contribute to the formation of esterified OPDA after wounding induced by a freeze-thaw cycle. The Col-0 allele of *HPL1* carries a deletion that creates a frameshift mutation and subsequently a premature stop codon (Duan *et al.*, 2005). Expression of *HPL1* was found to differ considerably between the accessions C24 and L*er* (Fig. 7) and we propose that this contributes to their difference in ability to produce arabidopsides. Although expression was also detectable in Col-0, no functional gene product is expected due to the frameshift mutation (Duan *et al.*, 2005). Snoeren *et al.* (2010) found only small differences in *HPL1* expression between Col-0, C24 and L*er* in their study of herbivore-induced volatiles in different Arabidopsis accessions. There are no apparent reasons to this difference in results, as growth conditions and age of the plants at tissue sampling were similar in both studies. However, in their study, among the nine accessions that were analyzed for emission of volatiles, at basal growth condition C24 showed the highest release of (*Z*)-3-hexen-1-ol, a product downstream of HPL1, suggesting high HPL1 activity.

When the C24 *HPL1* allele containing its native promoter was transformed into Col-0 plants, only a moderate increase in *HPL1* transcripts was observed and the levels of esterified OPDA remained unchanged as compared to control plants. This suggests that the very high abundance of *HPL1* transcripts in C24 could be attributed to accession-specific *trans*-acting elements. For this reason, we transformed Col-0 plants with a construct containing the coding sequence of the C24 *HPL1* fused to the strong constitutive CaMV 35S promoter. This resulted in high expression of the gene and consequently also reduced levels of lipid-bound OPDA (Fig. 8). The level of *HPL1* transcripts was directly proportional to the formation of OPDA esters (Fig. 8C). In support of this, it was previously reported that overexpression of rice *HPL* in the Col-0 background caused a reduction of free jasmonates and a substantial increase in C_6_-aldehydes in response to mechanical wounding (Chehab *et al.*, 2008). The elevated levels of C_6_-aldehydes greatly exceeded the loss of free jasmonates, suggesting that not only free fatty acids but also lipid bound hydroperoxy fatty acids were subjected to the increased HPL activity.

### Galactoplid bound hydroperoxides as substrate for CYP74 members

AOS, HPL and DES constitute an atypical subfamily (CYP74) of cytochrome P450 monooxygenases that neither requires molecular oxygen nor NADPH-reductase for their activity (Lee *et al.*, 2008, Hughes *et al.*, 2009). Their unusual reaction mechanism gives the enzymes an extraordinarily high turnover rate.

Arabidopside synthesis is independent of free fatty intermediates (Nilsson *et al.*, 2012). The formation is initiated by the enzymatic oxygenation of lipid bound fatty acids by LOX2 (Glauser *et al.*, 2009) followed by the conversion to an allene oxide by AOS (Park *et al.*, 2002). In this report we show that also the last step in the synthesis of galactolipid-bound (dn)OPDA is enzymatically catalyzed in response to freeze-thaw wounding. Thus, the rate of arabidopside formation is solely dependent on enzyme mediators after tissue damage. It still remains to be tested if one or all isoforms of AOC are involved in the synthesis of arabidopsides.

A recent study reported the presence of MGDG with acylated 7-OH-dinortraumatin and/or 9-OH-traumatin in wounded tissue from the Arabidopsis accessions L*er* and No-0 (Nakashima *et al.*, 2013). A concomitant increase in C_6_-aldehydes was also detected. The L*er* ecotype backcrossed to carry the malfunctional Col-0 *HPL1* allele did not accumulate traumatin-containing MGDGs in response to tissue disruption. It was also found that a L*er* line carrying a loss of function allele of *AOS* contained more MGDG with 7-OH-dinortraumatin and/or 9-OH-traumatin than the wild-type line, indicating substrate competition for lipid bound hydroperoxy fatty acids between AOS and HPL. Interestingly, the authors could detect significant amounts of MGDG containing 7-OH-dinortraumatin and/or 9-OH-traumatin after tissue disruption in several plant species that are reportedly unable to synthesize arabidopsides. Furthermore, synthesis of the majority of the six carbon volatiles via HPL in Arabidopsis have been shown to be dependent on LOX2 activity (Mochizuki *et al.*, 2016). Taken together, this and other studies provide strong evidence that HPL can access lipid-bound hydroperoxides fatty acids in a number of plant species.

MGDG and DGDG with divinyl ethers esters are known to accumulate in response to pathogen elicitation in flax (*Linum usitatissimum*) leaves (Chechetkin *et al.*, 2009). The synthesis of galactolipids bound divinyl ethers is controlled by DES activity. Notably, although free OPDA are present in the leaves of flax, no arabidopsides or other lipids with OPDA esters could be detected (Chechetkin *et al.*, 2009). The authors propose a biosynthesis mechanism where free fatty divinyl ethers acids are esterified to galactolipids. It thus seems like that two parallel biosynthesis pathways are involved in the formation of oxylipin-esters: one that requires enzymatic hydrolysis of lipid bound fatty acid, as in the case of esters of divinyl ethers in flax, and one pathway without any free fatty acid intermediates illustrated by the production of arabidopsides (Nilsson *et al.*, 2012) and lipid bound traumatin in Arabidopsis (Nakashima *et al.*, 2013).

HPL and AOS are genetically and structurally closely related. Replacing a single amino acid in the active site of AOS is sufficient to convert it into a hydroperoxide lyase that can produce green leaf volatiles (Lee *et al.*, 2008). Considering this, and the notion that HPL activity on bound hydroperoxy fatty acids appears to be fairly common in the plant kingdom, it is rather surprising that so few plant species appear to produce arabidopsides. Several hypotheses may be put forward to explain this, for example: (i) that bound 12, 13-EOT/10, 11-EHT or (dn)OPDA are usually released by lipases quickly after their formation, (ii) that plant species able to form arabidopsides hold a ‘special’ type of AOS that is active on glycerolipid bound substrates, or (iii) that arabidopside-producing plant species contain some co-factor that facilitates AOS activity on bound substrates and thus favors the jasmonate pathway over the HPL pathway.

In conclusion, we show that the ability to produce arabidopsides is conserved but varies within the Arabidopsis species. The results presented strengthen the idea that HPL1 negatively influences arabidopside formation by consuming galactolipid-bound hydroperoxide fatty acids generated through the activity of LOX2. We propose that differential expression of *HPL1* in different Arabidopsis accessions plays a central role in regulating arabidopside abundance. Finally, we present evidence that the last step in arabidopside synthesis, cyclization of 12, 13-EOT/10, 11-EHT, is enzymatic and most likely involves one or several AOCs. It still remains to be investigated how enzymes like HPL, LOX, AOS and AOC can gain access to and modify esterified fatty acids. The underlying mechanism to the very high expression level of *HPL1* in C24 also remains to be explained.

## Supplementary data

Supplementary data are available at *JXB* online.

Fig. S1. Alignment of coding sequences of Col-0 and C24 *HPL1*.

Fig. S2. Fine mapping of the QTL determining arabidopside accumulation.

Table S1. Quantification of galactolipid species before and 60 minutes after freeze-thaw in Arabidopsis accessions.

Table S2. List of primers used for qPCR.

Table S3. List of primers used for genetic mapping.

Supplementary Data
