# Supplementary material for: The activity of HYDROPEROXIDE LYASE 1 regulates accumulation of galactolipids containing 12-oxo-phytodienoic acid in Arabidopsis
Source: J Exp Bot. 2016 Jul 15;67(17):5133–44. doi: 10.1093/jxb/erw278 (PMC5014160; doi:10.1093/jxb/erw278)
Supplement: Supplementary Data [file supp_67_17_5133__index.html]

The activity of HYDROPEROXIDE LYASE 1 regulates accumulation of galactolipids containing 12-oxo-phytodienoic acid in Arabidopsis — The activity of HYDROPEROXIDE LYASE 1 regulates accumulation of galactolipids containing 12-oxo-phytodienoic acid in Arabidopsis — Supplementary Data 

# The activity of HYDROPEROXIDE LYASE 1 regulates accumulation of galactolipids containing 12-oxo-phytodienoic acid in Arabidopsis

## Supplementary Data

Data files

- supplementary\_figures\_S1\_S2\_Tables\_S1\_S3.pdf - Supplementary Data
