# Supplementary material for: The activity of HYDROPEROXIDE LYASE 1 regulates accumulation of galactolipids containing 12-oxo-phytodienoic acid in Arabidopsis
Source: J Exp Bot. 2016 Jul 15;67(17):5133–44. doi: 10.1093/jxb/erw278 (PMC5014160; doi:10.1093/jxb/erw278)
Supplement: Supplementary Data [file supp_erw278_supplementary_figures_S1_S2_Tables_S1_S3.pdf]

SUPPLEMENTARY MATERIAL

The activity of Hydroperoxide lyase 1 regulates accumulation of galactolipids containing 12-oxo-phytodienoic acid in *Arabidopsis thaliana*. Anders K Nilsson, Per Fahlberg, Oskar N Johansson, Mats Hamberg, Mats X Andersson and Mats Ellerström

```
Col-0        ATGTTGTTGAGAACGATGGCGGCGACTTCCCCGCGGCCACCACCGTCAACATCCCTAACATCTCAGCAGCCACCATCACCCCCCTCACAGCTTCCCCTCCGTACAATGCCGGGATCGTAC
C24          ATGTTGTTGAGAACGATGGCGGCGACTTCCCCGCGGCCACCACCGTCAACATCCCTAACATCTCAGCAGCCACCATCACCCCCCTCACAGCTTCCCCTCCGTACAATGCCGGGATCGTAC
             ************************************************************************************************************************

Col-0        GGCTGGCCGTTGGTTGGACCATTATCGGACCGTTTAGA----------TTCCAAGGACCCGATAAGTTTTTCCGGACAAGAGCTGAGAAGTATAAGAGCACTGTGTTCCGTACAAATATT
C24          GGCTGGCCGTTGGTTGGACCATTATCGGACCGTTTAGATTACTTCTGGTTCCAAGGACCCGATAAGTTTTTCCGGACAAGAGCTGAGAAGTATAAGAGCACTGTGTTCCGTACAAATATT
             **************************************          ************************************************************************

Col-0        CCTCCGACGTTTCCTTTCTTCGGCAACGTTAACCCTAACATCGTCGCCGTTCTTGACGTCAAGTCTTTTAGCCATCTTTTTGACATGGATCTAGTTGATAAAAGAGATGTTCTCATCGGA
C24          CCTCCGACGTTTCCTTTCTTCGGCAACGTTAACCCTAACATCGTCGCCGTTCTTGACGTCAAGTCTTTTAGCCATCTTTTTGACATGGATCTAGTTGATAAAAGAGATGTTCTCATCGGA
             ************************************************************************************************************************

Col-0        GACTTCCGGCCTAGCCTTGGGTTCTACGGCGGCGTTCGTGTTGGAGTTTATCTCGACACTACTGAGCCAAAGCACGCCAAGATAAAAGGTTTCGCTATGGAAACACTAAAACGAAGCTCA
C24          GACTTCCGGCCTAGCCTTGGGTTCTACGGCGGCGTTCGTGTTGGAGTTTATCTCGACACTACTGAGCCAAAGCACGCCAAGATAAAAGGTTTCGCTATGGAAACACTAAAACGAAGCTCA
             ************************************************************************************************************************

Col-0        AAAGTATGGCTACAAGAGCTTCGTTCAAACCTAAACATTTTCTGGGGAACAATCGAATCCGAAATCTCCAAAAACGGTGCCGCTTCATATATCTTCCCTCTCCAACGTTGCATCTTCAGT
C24          AAAGTATGGCTACAAGAGCTTCGTTCAAACCTAAACATTTTCTGGGGAACAATCGAATCCGAAATCTCCAAAAACGGTGCCGCTTCATATATCTTCCCTCTCCAACGTTGCATCTTCAGT
             ************************************************************************************************************************

Col-0        TTCCTCTGCGCCTCTCTCGCCGGCGTTGACGCTTCCGTATCGCCGGACATCGCTGAGAACGGTTGGAAAACAATCAATACTTGGCTTGCGTTGCAAGTTATTCCCACTGCTAAACTTGGC
C24          TTCCTCTGCGCCTCTCTCGCCGGCGTTGACGCTTCCGTATCGCCGGACATCGCTGAGAACGGTTGGAAAACAATCAATACTTGGCTTGCGTTGCAAGTTATTCCCACTGCTAAACTTGGC
             ************************************************************************************************************************

Col-0        GTAGTTCCTCAGCCTCTTGAAGAGATTTTACTTCATACTTGGCCTTATCCTTCTCTCTTAATCGCCGGAAATTACAAAAAGCTTTACAATTTCATCGACGAGAACGCCGGAGATTGTCTC
C24          GTAGTTCCTCAGCCTCTTGAAGAGATTTTACTTCATACTTGGCCTTATCCTTCTCTCTTAATCGCCGGAAATTACAAAAAGCTTTACAATTTCATCGACGAGAACGCCGGAGATTGTCTC
             ************************************************************************************************************************

Col-0        CGGTTAGGTCAAGAAGAATTCGGGTTGACCCGAGATGAGGCTATTCAAAATCTTCTCTTTGTTTTAGGTTTTAATGCCTACGGGGGCTTTTCCGTCTTCTTACCTTCTTTGATCGGGAGA
C24          CGGTTAGGTCAAGAAGAATTCGGGTTGACCCGAGATGAGGCTATTCAAAATCTTCTCTTTGTTTTAGGTTTTAATGCCTACGGGGGCTTTTCCGTCTTCTTACCTTCTTTGATCGGGAGA
             ************************************************************************************************************************

Col-0        ATAACCGGCGACAATTCCGGTTTACAGGAGAGGATTAGAACCGAAGTCAGGAGAGTTTGCGGATCCGGGTCGGATCTTAATTTCAAGACGGTTAACGAAATGGAGCTGGTTAAATCCGTG
C24          ATAACCGGCGACAATTCCGGTTTACAGGAGAGGATTAGAACCGAAGTCAGGAGAGTTTGCGGATCCGGGTCGGATCTTAATTTCAAGACGGTTAACGAAATGGAGCTGGTTAAATCCGTG
             ************************************************************************************************************************


Col-0        GTTTACGAAACGCTGCGTTTTAGTCCTCCGGTTCCGCTGCAATTCGCACGTGCGAGGAAAGATTTTCAGATAAGTTCACACGATGCTGTTTTTGAGGTCAAGAAAGGTGAGCTTCTTTGT
C24          GTTTACGAAACGCTGCGTTTTAGTCCTCCGGTTCCGCTGCAATTCGCACGTGCGAGGAAAGATTTTCAGATAAGTTCACACGATGCTGTTTTTGAGGTCAAGAAAGGTGAGCTTCTTTGT
```

```
             ************************************************************************************************************************

Col-0        GGTTATCAGCCGCTTGTGATGAGAGACGCTAATGTTTTTGACGAACCGGAGGAATTTAAACCGGACCGGTATGTAGGTGAGACCGGGTCTGAATTGCTGAATTATCTCTACTGGTCTAAC
C24          GGTTATCAGCCGCTTGTGATGAGAGACGCTAATGTTTTTGACGAACCGGAGGAATTTAAACCGGACCGGTATGTAGGTGAGACCGGGTCTGAATTGCTGAATTATCTCTACTGGTCTAAC
             ************************************************************************************************************************

Col-0        GGTCCACAAACCGGTACCCCGAGCGCGTCGAACAAACAGTGTGCAGCTAAGGACATTGTCACTCTCACGGCTTCCTTGCTCGTTGCCGATTTATTTCTCCGGTATGATACGATTACTGGT
C24          GGTCCACAAACCGGTACCCCGAGCGCGTCGAACAAACAGTGTGCAGCTAAGGACATTGTCACTCTCACGGCTTCCTTGCTCGTTGCCGATTTATTTCTCCGGTATGATACGATTACTGGT
             ************************************************************************************************************************

Col-0        GACTCCGGTTCAATTAAAGCTGTTGTTAAAGCTAAATAA
C24          GACTCCGGTTCAATTAAAGCTGTTGTTAAAGCTAAATAA
             ***************************************
```

Fig. S1. Alignment of the coding sequences of Col-0 and C24 *HPL1 alleles*. Highlighted in green is the transcriptional start codon suggested by Duan, Huang et al. (2005), red stop codon, dashed line indicates the 10 bp deletion found in Col-0. Col-0 genetic sequence was downloaded from TAIR (www.arabidopsis.org) and C24 sequence was determined by sequencing cDNA. Alignment was made using Clustal Omega (http://www.ebi.ac.uk/Tools/msa/clustalo/).

A

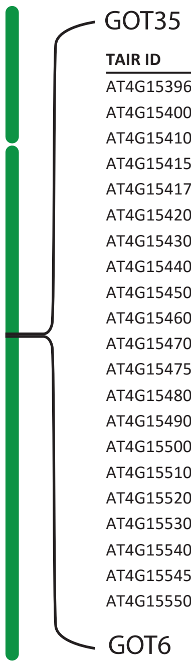

TAIR ID	Gene	Description	Localization	SNPs	NSS	NSSP	T-DNA line
AT4G15396	CYP702A6	A member of the cytochrome P450 family	Secreted[1]/Membrane[2]	22	7	3	SALK_202538C
AT4G15400	ABS1, BIA1	Encodes BIA1, a member of the BAHD acyltransferase family	Cytoplasm[2]	8	2	1	
AT4G15410	PUX5	Serine/threonine protein phosphatase 2A 55 kDa regulatory subunit B prime gamma	Nucleus[2]	3	1	0	SALK_115978C
AT4G15415	ATB' GAMMA	B' regulatory subunit of protein phosphatase 2A	Chloroplast[1]/Nucleus[2]	1	1	1	SALK_039172C
AT4G15417	RTL1	RNAse II-like 1	Cytoplasm[2]	2	1	1	
AT4G15420		Ubiquitin fusion degradation UFD1 family protein	Nucleus[2]	1	1	1	SALK_063032C
AT4G15430		ERD (early-responsive to dehydration stress) family protein	Secreted[1]/Membrane[2]	18	7	4**	SALK_058350, SALK_015653
AT4G15440	HPL1	Encodes a hydroperoxide lyase. A member of the CYP74B cytochrome p450 family	Chloroplast[2]	*	-	-	
AT4G15450		Senescence/dehydration-associated protein-related	Mitochondrion[1]	3	2	0	
AT4G15460		Glycine-rich protein	Secreted[1]/Membrane[2]	1	0	0	
AT4G15470		Bax inhibitor-1 family protein	Unknown[1]	2	1	0	SAIL_151_F11
AT4G15475		F-box/RNI-like superfamily protein	Nucleus	26	5	4	SALK_041396C
AT4G15480	UGT84A1	Encodes a protein that might have sinapic acid:UDP-glucose glucosyltransferase activity	Unknown[1]	44	13	8	SALK_129336C
AT4G15490	UGT84A3	Encodes a protein that might have sinapic acid:UDP-glucose glucosyltransferase activity	Unknown[1]	7	5	2	SAIL_75_C11, SALK_045492
AT4G15500	UGT84A4	Encodes a protein that might have sinapic acid:UDP-glucose glucosyltransferase activity	Unknown[1]	1	0	0	
AT4G15510	PPD1	A nuclear-encoded thylakoid lumenal protein that is associated with photosystem I (PSI)	Chloroplast[1, 2]	0	0	0	
AT4G15520		tRNA/rRNA methyltransferase (SpoU) family protein	Cytoplasm[2]	0	0	0	
AT4G15530	PPDK	Encodes a dual-targeted protein believed to act as a pyruvate, orthophosphate dikinase	Chloroplast[2]	2	1	0	SALK_023320
AT4G15540	UMAMIT38	Nodulin MtN21-like transporter family protein	Membrane[2]	3	2	1	SALK_050293C
AT4G15545			Unknown[1]	1	0	0	
AT4G15550	IAGLU	UDP-glucose:indole-3-acetate beta-D-glucosyltransferase	Chloroplast[1]/Cytoplasm[2]	1	1	1	

B

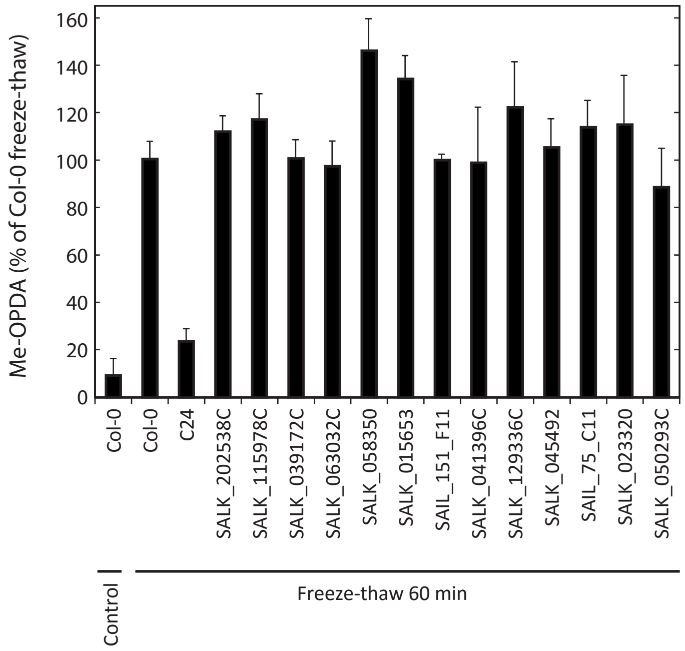

Fig. S2. Fine mapping of the QTL determining arabidopside accumulation. A, The QTL accountable for the difference in arabidopside accumulation between Col-0 and C24 could be isolated to a 64 kpb region on chromosome four flanked by the genetic markers GOT35 and GOT6. SNPs, number of single nucleotide polymorphisms; NSS, number of non-synonymous amino acids substitutions; NSSP, number of non-synonymous amino acids substitutions with altered chemical properties. Sub-cellular localization of gene products was retrieved from 1 TAIR (Lamesch et al. 2012) or predicted by 2 TargetP (Emanuelsson et al. 2000). * denotes a frameshift mutation in Col-0 and ** a premature stop codon in C24. **B**, A total lipid extract derived from leaf discs from Col-0, C24, or homozygous T-DNA mutant plants in Col-0 genetic background were transmethylated and analyzed for glycerolipid bound OPDA by GC-MS. Data was normalized to the level of Me-OPDA in Col-0 after 60 minutes of freeze-thaw treatment. Error bars represent standard deviation where n=3-6.

Table S1. Quantification of galactolipid species before and 60 minutes after freeze-thaw in Arabidopsis accessions. Letter after lipid species indicate standard used for quantification (see material and methods), a) MGDG, b) DGDG, c) tri18:3-acyl-MGDG, d) Arabidopside A, e) Arabidopside E.

Lipid species	M[NH4]$^+$ (*m/z*)	Neutral loss	Product ion	Interpreted fatty acid composition	Trivial name	Treatment	C 24	Ws-0	No-0	Uk-4	Kas-1	Ler	Po-0	Sei-0	Li-0	Nd-0	Ts-U	Per-1	Mo-2	Col-0
34:3-MG [a]	770.7	MG	591.6	18:3, 16:0		Control	28.2 ± 1.4	26.7 ± 1.4	34.1 ± 2.0	28.4 ± 3.7	26.4 ± 2.6	29.8 ± 2.4	29.2 ± 2.7	30.5 ± 1.3	19.7 ± 2.6	32.1 ± 3.2	28.4 ± 1.0	34.6 ± 1.9	30.8 ± 1.8	31.0 ± 3.0
						Freeze-thaw	3.7 ± 0.7	2.8 ± 0.3	3.6 ± 0.9	4.3 ± 0.8	1.6 ± 0.1	2.9 ± 0.5	2.4 ± 0.2	1.3 ± 0.1	2.0 ± 0.7	2.8 ± 0.6	2.8 ± 0.8	2.8 ± 0.4	1.7 ± 0.0	2.1 ± 0.4
34:4-MG [a]	768.7	MG	589.6	18:3, 16:1		Control	70.6 ± 3.4	57.8 ± 2.5	75.9 ± 1.7	56.6 ± 16.7	60.6 ± 3.9	59.1 ± 2.2	68.0 ± 1.0	76.7 ± 2.1	55.1 ± 4.8	71.8 ± 8.1	67.7 ± 4.2	86.1 ± 5.8	70.8 ± 5.6	80.5 ± 11.1
						Freeze-thaw	6.8 ± 1.3	4.1 ± 0.3	6.1 ± 1.3	6.5 ± 1.0	2.3 ± 0.3	3.9 ± 0.8	3.7 ± 0.3	1.7 ± 0.2	3.0 ± 1.1	3.6 ± 0.9	3.9 ± 1.3	3.0 ± 0.7	2.6 ± 0.2	2.9 ± 0.8
34:5-MG [a]	766.6	MG	587.5	18:3, 16:2		Control	94.4 ± 2.7	99.9 ± 2.5	134.3 ± 7.4	94.3 ± 5.9	99.4 ± 4.3	95.1 ± 10.6	110.9 ± 2.9	128.0 ± 3.6	68.8 ± 2.4	112.2 ± 17.4	96.5 ± 3.6	97.9 ± 8.6	103.5 ± 10.8	137.8 ± 23.1
						Freeze-thaw	4.1 ± 0.5	2.8 ± 0.2	3.2 ± 0.8	3.3 ± 0.5	1.3 ± 0.1	2.5 ± 0.6	1.8 ± 0.3	0.9 ± 0.0	1.3 ± 0.4	1.8 ± 0.6	1.7 ± 0.6	1.1 ± 0.6	1.6 ± 0.2	1.4 ± 0.5
34:6-MG [a]	764.6	MG	585.5	18:3, 16:3		Control	825.3 ± 3.6	827.7 ± 2.7	800.1 ± 13.1	874.3 ± 38.1	781.9 ± 12.6	817.8 ± 46.6	783.9 ± 2.2	801.5 ± 8.0	737.2 ± 3.3	783.7 ± 45.0	793.9 ± 9.8	821.2 ± 28.4	800.9 ± 33.4	886.1 ± 35.9
						Freeze-thaw	58.8 ± 11.8	27.1 ± 1.2	20.9 ± 5.5	48.8 ± 7.4	15.5 ± 1.4	32.7 ± 6.9	17.2 ± 2.8	8.7 ± 0.2	18.1 ± 4.5	19.4 ± 3.5	20.2 ± 6.6	15.1 ± 3.3	19.9 ± 1.3	11.5 ± 3.1
36:4-MG [a]	796.7	MG	617.6	18:3, 18:1		Control	37.7 ± 4.8	29.3 ± 1.2	34.8 ± 3.1	20.4 ± 14.1	22.5 ± 0.8	28.5 ± 2.0	31.9 ± 1.0	32.7 ± 1.0	17.4 ± 2.8	31.1 ± 2.1	34.9 ± 1.8	45.7 ± 4.0	37.1 ± 1.2	36.9 ± 3.4
						Freeze-thaw	3.1 ± 1.6	4.3 ± 0.5	4.1 ± 0.8	4.6 ± 0.8	2.2 ± 0.2	3.4 ± 0.6	3.4 ± 0.3	1.5 ± 0.7	2.5 ± 0.6	3.6 ± 0.6	3.1 ± 1.9	4.0 ± 0.5	2.8 ± 0.0	2.9 ± 0.4
36:5-MG [a]	794.7	MG	615.6	18:3, 18:2		Control	47.4 ± 5.4	50.9 ± 3.2	66.1 ± 4.2	35.7 ± 9.9	35.1 ± 0.3	45.3 ± 2.5	66.0 ± 7.9	61.5 ± 3.8	27.6 ± 2.5	53.2 ± 4.3	48.0 ± 2.5	53.6 ± 1.6	46.9 ± 2.1	58.9 ± 8.4
						Freeze-thaw	4.0 ± 0.7	3.5 ± 0.2	4.1 ± 0.9	4.1 ± 0.6	1.7 ± 0.1	3.4 ± 0.8	3.0 ± 0.4	1.8 ± 0.1	2.2 ± 0.4	3.1 ± 0.5	2.7 ± 0.6	2.3 ± 0.5	2.3 ± 0.0	2.4 ± 0.5
36:6-MG [a]	792.7	MG	613.6	18:3, 18:3		Control	1238.1 ± 15.4	1307.6 ± 35.3	1368.7 ± 41.0	1294.6 ± 67.4	1231.4 ± 31.5	1369.0 ± 65.3	1308.4 ± 46.7	1278.0 ± 34.9	928.3 ± 34.6	1259.0 ± 134.7	1332.6 ± 43.3	1389.6 ± 44.7	1276.1 ± 22.8	1230.7 ± 143.0
						Freeze-thaw	69.0 ± 9.8	49.8 ± 2.0	44.4 ± 10.7	81.0 ± 12.5	32.7 ± 1.6	60.6 ± 12.2	34.7 ± 2.7	22.6 ± 0.8	35.7 ± 9.1	49.4 ± 6.8	43.1 ± 10.5	37.3 ± 8.0	40.6 ± 0.9	27.8 ± 4.5
34:3-DG [b]	932.8	DG	591.3	18:3, 16:0		Control	72.4 ± 2.0	100.2 ± 4.6	86.3 ± 3.6	105.5 ± 7.2	83.8 ± 5.0	93.5 ± 6.8	86.3 ± 8.8	85.3 ± 4.5	65.0 ± 6.1	81.8 ± 5.8	90.3 ± 5.5	98.2 ± 3.8	88.8 ± 0.4	70.9 ± 6.1
						Freeze-thaw	17.6 ± 2.8	19.3 ± 1.3	13.8 ± 3.8	21.0 ± 3.9	8.0 ± 0.5	15.4 ± 1.3	9.0 ± 1.3	4.8 ± 0.3	7.5 ± 2.0	9.1 ± 1.7	11.0 ± 2.8	8.7 ± 1.1	8.6 ± 0.5	6.5 ± 1.5
34:4-DG [b]	930.8	DG	589.3	18:3, 16:1		Control	3.8 ± 0.0	4.1 ± 0.1	3.9 ± 0.4	4.2 ± 0.5	2.4 ± 1.9	4.1 ± 0.4	3.7 ± 0.1	1.7 ± 2.4	2.9 ± 0.2	3.9 ± 0.3	4.1 ± 0.2	4.8 ± 0.5	3.2 ± 2.5	4.0 ± 0.5
						Freeze-thaw	0.5 ± 0.4	0.5 ± 0.4	0.5 ± 0.1	0.4 ± 0.3	0.3 ± 0.0	0.6 ± 0.1	0.3 ± 0.1	0.2 ± 0.0	0.3 ± 0.1	0.4 ± 0.1	0.2 ± 0.2	0.3 ± 0.1	0.4 ± 0.0	0.3 ± 0.1
34:5-DG [b]	928.8	DG	587.3	18:3, 16:2		Control	4.8 ± 4.8	4.9 ± 4.9	4.9 ± 4.9	4.0 ± 4.0	3.3 ± 3.3	4.3 ± 4.3	3.9 ± 3.9	5.6 ± 5.6	3.2 ± 3.2	4.6 ± 4.6	4.2 ± 4.2	3.6 ± 3.6	4.4 ± 4.4	5.9 ± 5.9
						Freeze-thaw	0.9 ± 0.0	0.7 ± 0.0	0.6 ± 0.1	0.5 ± 0.1	0.3 ± 0.0	0.6 ± 0.1	0.3 ± 0.1	0.2 ± 0.0	0.3 ± 0.1	0.3 ± 0.1	0.4 ± 0.1	0.2 ± 0.1	0.4 ± 0.0	0.4 ± 0.1
34:6-DG [b]	926.8	DG	585.3	18:3, 16:3		Control	36.5 ± 1.6	41.6 ± 1.4	30.5 ± 1.1	42.3 ± 5.1	28.9 ± 0.7	37.8 ± 6.3	28.9 ± 0.2	36.1 ± 1.0	29.4 ± 1.3	33.1 ± 2.5	35.4 ± 2.2	35.2 ± 2.3	35.2 ± 2.1	39.2 ± 2.1
						Freeze-thaw	3.7 ± 0.4	2.4 ± 0.1	1.5 ± 0.3	2.1 ± 0.3	1.1 ± 0.0	2.2 ± 0.4	1.0 ± 0.2	0.7 ± 0.1	1.1 ± 0.2	1.2 ± 0.1	1.3 ± 0.4	0.8 ± 0.2	1.5 ± 0.2	0.9 ± 0.2
34:4-DG [b]	958.8	DG	617.3	18:3, 18:1		Control	6.3 ± 0.2	7.0 ± 0.3	6.7 ± 0.3	6.9 ± 1.6	6.0 ± 0.1	6.5 ± 0.4	6.5 ± 0.3	6.3 ± 0.1	4.2 ± 0.3	5.9 ± 0.3	7.1 ± 0.3	9.5 ± 0.7	8.4 ± 0.5	5.2 ± 0.4
						Freeze-thaw	1.9 ± 0.2	1.9 ± 0.1	1.5 ± 0.3	1.7 ± 0.3	0.9 ± 0.0	1.7 ± 0.1	1.1 ± 0.1	0.7 ± 0.1	0.8 ± 0.1	1.2 ± 0.1	1.4 ± 0.3	1.3 ± 0.1	1.3 ± 0.1	0.6 ± 0.5
36:5-DG [b]	956.8	DG	615.3	18:3, 18:2		Control	9.5 ± 0.5	10.9 ± 0.4	12.3 ± 0.5	7.3 ± 1.1	7.3 ± 0.2	9.0 ± 0.9	12.8 ± 1.1	13.6 ± 0.5	6.5 ± 0.3	10.7 ± 0.9	9.7 ± 0.2	9.0 ± 0.7	9.8 ± 0.6	11.6 ± 1.1
						Freeze-thaw	1.8 ± 0.1	2.1 ± 0.1	1.6 ± 0.4	1.1 ± 0.1	0.7 ± 0.0	1.5 ± 0.2	1.0 ± 0.1	0.8 ± 0.1	0.6 ± 0.1	1.1 ± 0.2	0.9 ± 0.2	0.6 ± 0.3	1.0 ± 0.0	1.0 ± 0.2
36:6-DG [b]	954.8	DG	613.3	18:3, 18:3		Control	493.4 ± 20.2	543.6 ± 15.5	532.2 ± 12.7	533.3 ± 30.4	493.6 ± 11.4	519.8 ± 22.6	539.2 ± 21.4	543.3 ± 8.8	419.7 ± 30.0	516.8 ± 30.5	544.0 ± 15.8	568.6 ± 30.3	500.7 ± 17.6	508.9 ± 19.0
						Freeze-thaw	87.9 ± 9.2	70.3 ± 1.6	49.5 ± 12.9	63.6 ± 10.7	34.1 ± 2.3	69.3 ± 13.8	35.6 ± 4.6	23.5 ± 1.3	29.7 ± 4.8	40.3 ± 7.1	42.8 ± 9.2	33.6 ± 5.9	44.2 ± 1.8	30.3 ± 6.3
34:6-MG-16:0 [c]	1002.9	MG-16:0	585.4	18:3, 16:3, 16:0		Control	0.3 ± 0.2	0.0 ± 0.0	0.0 ± 0.0	0.0 ± 0.0	0.1 ± 0.0	0.1 ± 0.1	0.0 ± 0.0	0.1 ± 0.0	0.0 ± 0.0	0.3 ± 0.3	0.1 ± 0.0	0.1 ± 0.0	0.2 ± 0.1	0.0 ± 0.0
						Freeze-thaw	19.0 ± 3.3	9.1 ± 1.1	5.0 ± 0.8	2.3 ± 0.2	6.5 ± 0.8	10.2 ± 1.4	2.3 ± 0.8	2.9 ± 1.7	2.7 ± 0.4	3.6 ± 0.8	2.1 ± 1.0	0.9 ± 0.2	11.4 ± 2.5	2.7 ± 1.2
34:6-MG-16:3 [c]	996.8	MG-16:3	585.4	18:3, 16:3, 16:3		Control	0.1 ± 0.1	0.0 ± 0.0	0.0 ± 0.0	0.0 ± 0.0	0.0 ± 0.0	0.1 ± 0.0	0.0 ± 0.0	0.0 ± 0.0	0.0 ± 0.0	0.1 ± 0.1	0.0 ± 0.0	0.0 ± 0.0	0.1 ± 0.0	0.0 ± 0.0
						Freeze-thaw	15.0 ± 4.6	4.8 ± 1.5	2.5 ± 0.2	1.6 ± 0.1	5.0 ± 0.6	7.8 ± 1.6	1.4 ± 0.7	2.0 ± 1.4	2.0 ± 0.6	2.2 ± 0.6	1.2 ± 0.8	0.3 ± 0.2	11.6 ± 3.5	1.6 ± 0.7
34:6-MG-18:3 [c]	1024.8	MG-18:3	585.4	18:3, 16:3, 18:3		Control	4.0 ± 3.6	0.6 ± 0.1	0.6 ± 0.2	0.6 ± 0.4	0.9 ± 0.5	1.8 ± 0.6	0.4 ± 0.0	1.2 ± 0.3	0.6 ± 0.2	2.5 ± 2.4	0.8 ± 0.1	1.1 ± 0.5	2.9 ± 1.0	0.6 ± 0.1
						Freeze-thaw	98.5 ± 21.5	46.0 ± 8.7	25.4 ± 2.9	16.2 ± 1.7	41.6 ± 6.2	53.8 ± 1.8	16.2 ± 6.4	21.3 ± 11.0	18.3 ± 4.4	21.5 ± 5.0	14.7 ± 7.6	5.6 ± 0.9	79.1 ± 22.6	16.7 ± 6.8
36:6-MG-18:3 [c]	1052.9	MG-18:3	613.5	18:3, 18:3, 18:3		Control	3.1 ± 2.5	0.6 ± 0.1	0.5 ± 0.5	0.4 ± 0.7	1.1 ± 0.6	2.3 ± 1.0	0.4 ± 0.1	1.1 ± 0.3	0.5 ± 0.2	2.9 ± 2.7	0.8 ± 0.2	1.3 ± 0.6	2.6 ± 0.9	0.4 ± 0.4
						Freeze-thaw	91.6 ± 21.2	57.4 ± 4.8	41.0 ± 4.6	21.1 ± 1.8	60.2 ± 9.8	86.2 ± 11.1	23.1 ± 6.4	31.7 ± 14.6	26.9 ± 1.9	39.4 ± 5.1	28.8 ± 10.7	17.3 ± 2.6	99.0 ± 14.9	27.3 ± 8.2
34:4-O-MG [d]	784.7	MG	605.5	OPDA, 16:0		Control	0.1 ± 0.0	0.0 ± 0.0	0.1 ± 0.0	0.1 ± 0.1	0.0 ± 0.0	0.1 ± 0.1	0.1 ± 0.1	0.1 ± 0.0	0.0 ± 0.0	0.1 ± 0.2	0.1 ± 0.0	0.2 ± 0.1	0.1 ± 0.1	0.1 ± 0.0
						Freeze-thaw	0.3 ± 0.1	0.4 ± 0.2	0.7 ± 0.1	1.8 ± 0.5	0.6 ± 0.1	0.9 ± 0.1	0.9 ± 0.3	0.4 ± 0.0	1.1 ± 0.3	1.4 ± 0.3	1.4 ± 0.1	2.5 ± 0.7	0.7 ± 0.2	0.8 ± 0.1
34:5-O-MG [d]	782.6	MG	603.5	OPDA, 16:1		Control	0.0 ± 0.0	0.0 ± 0.0	0.0 ± 0.0	0.0 ± 0.1	0.0 ± 0.0	0.0 ± 0.0	0.1 ± 0.1	0.0 ± 0.0	0.0 ± 0.0	0.0 ± 0.1	0.0 ± 0.0	0.1 ± 0.1	0.0 ± 0.0	0.0 ± 0.0
						Freeze-thaw	0.5 ± 0.1	0.4 ± 0.1	0.5 ± 0.0	0.8 ± 0.2	0.5 ± 0.1	0.6 ± 0.1	0.7 ± 0.1	0.6 ± 0.0	1.2 ± 0.1	1.3 ± 0.2	1.4 ± 0.2	3.2 ± 1.1	0.8 ± 0.2	1.3 ± 0.2
34:6-O-MG [d]	780.6	MG	601.5	OPDA, 16:2		Control	0.1 ± 0.0	0.1 ± 0.0	0.1 ± 0.0	0.1 ± 0.0	0.0 ± 0.0	0.1 ± 0.0	0.1 ± 0.1	0.1 ± 0.0	0.1 ± 0.0	0.2 ± 0.2	0.0 ± 0.0	0.1 ± 0.0	0.0 ± 0.0	0.1 ± 0.0
						Freeze-thaw	0.4 ± 0.3	0.4 ± 0.3	0.5 ± 0.4	0.1 ± 0.1	0.3 ± 0.2	0.4 ± 0.3	0.1 ± 0.0	0.0 ± 0.0	0.3 ± 0.3	0.1 ± 0.0	0.1 ± 0.0	0.1 ± 0.0	0.4 ± 0.2	0.3 ± 0.4
34:7-O-MG [d]	778.6	MG	599.5	OPDA, 16:3	MDGD-O	Control	3.5 ± 2.6	0.6 ± 0.2	0.8 ± 0.4	2.1 ± 5.0	0.8 ± 0.2	4.0 ± 4.7	0.5 ± 0.3	1.6 ± 0.5	4.7 ± 4.6	12.7 ± 19.0	2.4 ± 1.1	7.8 ± 6.2	9.9 ± 8.6	1.3 ± 0.9
						Freeze-thaw	8.8 ± 1.4	6.9 ± 0.3	5.9 ± 1.6	1.1 ± 0.2	3.8 ± 0.5	7.2 ± 0.9	3.1 ± 2.3	2.7 ± 0.1	2.7 ± 1.7	3.7 ± 0.7	0.6 ± 0.1	1.4 ± 1.5	4.4 ± 0.1	2.7 ± 2.2
36:6-O-MG [d]	808.7	MG	629.5	OPDA, 18:2		Control	0.0 ± 0.0	0.0 ± 0.0	0.0 ± 0.0	0.0 ± 0.0	0.0 ± 0.0	0.1 ± 0.1	0.0 ± 0.0	0.0 ± 0.0	0.0 ± 0.0	0.0 ± 0.0	0.0 ± 0.0	0.0 ± 0.0	0.0 ± 0.0	0.1 ± 0.0
						Freeze-thaw	0.1 ± 0.1	0.0 ± 0.0	0.1 ± 0.2	0.2 ± 0.3	0.0 ± 0.0	0.1 ± 0.1	0.2 ± 0.2	0.0 ± 0.0	0.0 ± 0.0	0.0 ± 0.0	0.0 ± 0.0	0.1 ± 0.1	0.1 ± 0.1	0.0 ± 0.0
36:7-O-MG [d]	806.6	MG	627.5	OPDA, 18:3		Control	1.2 ± 0.8	0.3 ± 0.1	0.4 ± 0.2	0.6 ± 2.6	0.3 ± 0.0	2.0 ± 2.4	0.2 ± 0.1	0.7 ± 0.2	1.4 ± 1.5	5.8 ± 8.0	0.9 ± 0.3	3.5 ± 3.2	3.9 ± 3.6	0.5 ± 0.2
						Freeze-thaw	1.0 ± 0.2	0.9 ± 0.1	1.0 ± 0.3	2.0 ± 0.6	0.6 ± 0.1	1.4 ± 0.4	0.9 ± 0.1	0.4 ± 0.0	0.8 ± 0.1	0.8 ± 0.1	1.1 ± 0.4	0.8 ± 0.1	0.8 ± 0.1	0.5 ± 0.2
34:7-O-MG [d]	778.6	MG	599.5	dnOPDA, 18:3	Arabidopside F	Control	2.0 ± 0.6	1.6 ± 0.6	0.8 ± 0.2	0.5 ± 0.8	0.7 ± 0.3	1.5 ± 0.7	0.4 ± 0.2	0.9 ± 0.2	1.0 ± 0.6	12.4 ± 19.4	1.4 ± 0.3	1.8 ± 0.5	9.5 ± 9.2	1.7 ± 0.9
						Freeze-thaw	8.8 ± 1.4	6.8 ± 0.3	5.9 ± 1.6	1.1 ± 0.2	3.8 ± 0.5	7.1 ± 0.9	3.1 ± 2.3	2.6 ± 0.1	2.7 ± 1.7	3.7 ± 0.7	0.6 ± 0.1	1.5 ± 1.5	4.4 ± 0.1	2.7 ± 2.2
34:8-2O-MG-16:3 [e]	1024.8	MG-16:3	613.4	OPDA, dnOPDA, dnOPDA		Control	0.0 ± 0.0	0.0 ± 0.0	0.0 ± 0.0	0.0 ± 0.0	0.0 ± 0.0	0.0 ± 0.0	0.0 ± 0.0	0.0 ± 0.0	0.0 ± 0.0	0.0 ± 0.0	0.0 ± 0.0	0.0 ± 0.0	0.0 ± 0.0	0.0 ± 0.0
						Freeze-thaw	5.3 ± 0.3	5.6 ± 0.1	3.2 ± 0.4	2.6 ± 0.1	5.8 ± 0.3	8.5 ± 0.5	2.8 ± 0.6	3.0 ± 0.1	3.2 ± 0.2	3.2 ± 0.6	2.6 ± 0.8	0.9 ± 0.7	9.9 ± 0.1	4.2 ± 0.6
36:8-2O-MG-16:0 [e]	1058.9	MG-16:0	641.5	OPDA, OPDA, 16:0		Control	0.0 ± 0.0	0.0 ± 0.0	0.0 ± 0.0	0.0 ± 0.0	0.0 ± 0.0	0.0 ± 0.0	0.0 ± 0.0	0.0 ± 5.3	0.0 ± 0.0	0.0 ± 0.0	0.0 ± 0.0	0.0 ± 0.0	0.0 ± 0.0	0.0 ± 0.0
						Freeze-thaw	4.6 ± 0.1	7.9 ± 2.3	9.5 ± 0.1	10.9 ± 0.7	11.8 ± 1.2	17.6 ± 0.4	12.3 ± 0.4	9.8 ± 0.3	9.4 ± 0.4	17.1 ± 2.4	18.9 ± 0.8	21.4 ± 1.6	18.9 ± 3.0	16.1 ± 1.1
36:8-2O-MG-18:3 [e]	1080.8	MG-18:3	641.5	OPDA, OPDA, 18:3		Control	0.0 ± 0.0	0.0 ± 0.0	0.0 ± 0.0	0.0 ± 0.0	0.0 ± 0.0	0.0 ± 0.0	0.0 ± 0.0	0.0 ± 0.0	0.0 ± 0.0	0.0 ± 0.0	0.0 ± 0.0	0.0 ± 0.0	0.0 ± 0.0	0.0 ± 0.0
						Freeze-thaw	4.6 ± 0.1	6.0 ± 0.6	4.7 ± 0.6	2.7 ± 0.0	5.0 ± 0.2	9.1 ± 0.5	3.9 ± 0.4	3.0 ± 0.0	2.8 ± 0.0	4.5 ± 0.5	4.2 ± 0.7	1.8 ± 0.8	7.0 ± 0.4	4.0 ± 0.5
34:8-2O-MG-16:0 [e]	1030.8	MG-16:0	613.4	OPDA, dnOPDA, 16:0		Control	0.0 ± 0.0	0.0 ± 0.0	0.0 ± 0.0	0.1 ± 0.0	0.0 ± 0.0	0.1 ± 0.0	0.1 ± 0.0	0.1 ± 0.0	0.0 ± 0.0	0.1 ± 0.1	0.1 ± 0.1	0.1 ± 0.0	0.1 ± 0.1	0.1 ± 0.1
						Freeze-thaw	23.5 ± 1.3	49.2 ± 10.5	44.9 ± 0.2	50.0 ± 3.6	71.4 ± 6.1	91.1 ± 0.9	58.2 ± 3.0	70.9 ± 1.5	73.7 ± 2.5	95.5 ± 9.3	96.9 ± 7.7	92.3 ± 6.3	131.1 ± 14.0	114.3 ± 6.3
34:8-2O-MG-18:3 [e]	1052.8	MG-18:3	613.4	OPDA, dnOPDA, 18:3		Control	0.1 ± 0.0	0.1 ± 0.1	0.0 ± 0.0	0.1 ± 0.0	0.0 ± 0.0	0.2 ± 0.0	0.0 ± 0.0	0.0 ± 0.0	0.0 ± 0.0	0.1 ± 0.1	0.1 ± 0.1	0.1 ± 0.0	0.1 ± 0.0	0.2 ± 0.1
						Freeze-thaw	88.3 ± 4.5	103.1 ± 2.7	103.8 ± 27.2	49.7 ± 11.3	104.0 ± 12.7	174.0 ± 34.8	59.4 ± 1.1	54.5 ± 2.0	53.0 ± 8.7	51.4 ± 12.8	65.7 ± 27.0	21.0 ± 11.5	173.1 ± 32.5	71.6 ± 35.9
34:8-2O-MG-16:4-O [e]	1038.7	MG-16:4-O	613.4	OPDA, dnOPDA, dnOPDA		Control	0.0 ± 0.0	0.0 ± 0.0	0.0 ± 0.0	0.0 ± 0.0	0.0 ± 0.0	0.0 ± 0.0	0.0 ± 0.0	0.0 ± 0.0	0.0 ± 0.0	0.0 ± 0.0	0.0 ± 0.0	0.0 ± 0.0	0.0 ± 0.0	0.0 ± 0.0
						Freeze-thaw	0.3 ± 0.0	1.1 ± 0.5	1.1 ± 0.1	1.1 ± 0.2	2.4 ± 0.5	2.6 ± 0.2	1.8 ± 0.2	3.5 ± 0.1	3.0 ± 0.3	4.3 ± 0.6	4.7 ± 0.6	3.1 ± 0.6	5.7 ± 1.1	7.7 ± 1.2
34:8-2O-MG [d]	792.6	MG	613.4	OPDA, dnOPDA	Arabidopside A	Control	18.7 ± 4.2	20.9 ± 7.7	27.3 ± 10.2	53.2 ± 21.6	36.3 ± 10.2	52.6 ± 13.9	39.1 ± 2.2	49.6 ± 5.3	48.6 ± 4.9	72.2 ± 64.1	58.9 ± 6.7	83.1 ± 18.4	81.3 ± 25.5	64.8 ± 12.3
						Freeze-thaw	17.6 ± 8.3	19.3 ± 20.3	13.8 ± 13.8	21.0 ± 75.0	8.0 ± 18.0	15.4 ± 15.8	9.0 ± 24.2	4.8 ± 1.9	7.5 ± 44.7	9.1 ± 28.5	11.0 ± 32.4	8.7 ± 120.0	8.6 ± 37.5	6.5 ± 26.7
36:8-2O-MG [d]	820.6	MG	641.5	OPDA, OPDA	Arabidopside B	Control	2.7 ± 0.5	2.8 ± 1.2	4.2 ± 2.0	6.6 ± 5.4	4.8 ± 1.2	8.0 ± 2.8	6.3 ± 0.5	7.0 ± 1.2	5.5 ± 0.8	10.6 ± 9.2	9.6 ± 1.5	15.0 ± 4.2	10.5 ± 4.0	8.7 ± 1.9
						Freeze-thaw	3.4 ± 0.8	4.2 ± 2.3	8.5 ± 0.7	39.2 ± 9.8	8.4 ± 1.9	12.2 ± 1.7	18.7 ± 3.3	6.5 ± 0.2	17.9 ± 6.1	19.7 ± 3.4	26.4 ± 3.3	51.3 ± 20.6	10.5 ± 4.0	11.9 ± 2.7
34:8-2O-MG-18:4-O [e]	1066.8	MG-18:4-O	613.4	OPDA, dnOPDA, OPDA	Arabidopside E	Control	0.0 ± 0.0	0.0 ± 0.0	0.1 ± 0.1	0.4 ± 0.1	0.1 ± 0.1	0.1 ± 0.0	0.2 ± 0.1	0.3 ± 0.1	0.1 ± 0.1	0.4 ± 0.3	0.4 ± 0.3	0.6 ± 0.2	0.3 ± 0.2	0.5 ± 0.3
						Freeze-thaw	79.5 ± 2.8	172.5 ± 65.7	217.0 ± 21.4	310.6 ± 24.1	356.9 ± 29.9	402.4 ± 10.6	397.0 ± 16.1	447.8 ± 6.5	503.1 ± 19.4	535.5 ± 92.0	652.0 ± 35.5	646.6 ± 11.5	692.9 ± 94.3	698.9 ± 54.8
36:8-2O-MG-18:4-O [e]	1094.8	MG-18:4-O	641.5	OPDA, OPDA, OPDA	Arabidopside G	Control	0.0 ± 0.0	0.0 ± 0.0	0.0 ± 0.0	0.1 ± 0.0	0.0 ± 0.0	0.0 ± 0.0	0.0 ± 0.0	0.1 ± 0.0	0.0 ± 0.0	0.1 ± 0.0	0.1 ± 0.1	0.2 ± 0.1	0.0 ± 0.0	0.1 ± 0.1
						Freeze-thaw	12.0 ± 0.4	24.0 ± 12.5	42.8 ± 7.7	64.7 ± 5.1	61.4 ± 8.6	70.0 ± 2.5	84.5 ± 4.4	73.9 ± 2.1	81.8 ± 4.7	115.1 ± 29.9	154.4 ± 8.3	186.6 ± 4.9	130.6 ± 27.1	133.2 ± 16.6
34:8-2O-DG [d]	954.7	DG	613.4	OPDA, dnOPDA	Arabidopside C	Control	0.1 ± 0.0	0.1 ± 0.1	0.1 ± 0.1	0.2 ± 0.1	0.0 ± 0.0	0.4 ± 0.1	0.1 ± 0.1	0.1 ± 0.1	0.2 ± 0.2	0.1 ± 0.1	0.4 ± 0.3	0.2 ± 0.2	0.1 ± 0.1	0.6 ± 0.2
						Freeze-thaw	0.1 ± 0.1	0.1 ± 0.0	0.2 ± 0.0	2.4 ± 0.5	0.8 ± 0.2	0.7 ± 0.5	1.7 ± 0.4	1.1 ± 0.2	1.6 ± 0.8	1.8 ± 0.6	0.4 ± 0.1	3.2 ± 1.0	1.8 ± 0.7	0.9 ± 0.3
36:8-2O-DG [d]	982.8	DG	641.5	OPDA, OPDA	Arabidopside D	Control	0.6 ± 0.2	0.8 ± 0.5	1.0 ± 0.8	1.9 ± 1.3	1.1 ± 0.6	1.7 ± 0.5	1.5 ± 0.2	2.0 ± 0.5	1.7 ± 0.1	2.4 ± 1.8	2.9 ± 0.8	4.2 ± 0.4	2.7 ± 1.1	2.7 ± 0.8
						Freeze-thaw	1.2 ± 0.4	1.8 ± 1.1	3.8 ± 0.5	18.9 ± 5.3	5.0 ± 1.1	7.2 ± 0.6	12.1 ± 2.6	3.5 ± 0.2	12.7 ± 3.2	11.9 ± 4.6	15.3 ± 1.5	32.1 ± 12.9	7.1 ± 3.6	6.5 ± 2.1
34:4-O-DG [d]	946.8	DG	605.5	OPDA, 16:0		Control	0.1 ± 0.0	0.1 ± 0.1	0.1 ± 0.1	0.4 ± 0.1	0.1 ± 0.1	0.2 ± 0.1	0.2 ± 0.1	0.2 ± 0.1	0.2 ± 0.1	0.4 ± 0.3	0.3 ± 0.1	0.6 ± 0.1	0.3 ± 0.2	0.3 ± 0.1
						Freeze-thaw	0.8 ± 0.1	1.6 ± 0.7	1.8 ± 0.2	5.3 ± 1.3	2.0 ± 0.4	2.6 ± 0.2	3.3 ± 0.7	1.8 ± 0.1	3.8 ± 0.7	3.8 ± 1.0	4.7 ± 0.2	7.4 ± 2.3	3.0 ± 1.1	2.8 ± 0.5
36:7-O-DG [d]	968.8	DG	627.5	OPDA, 18:3		Control	0.4 ± 0.2	0.1 ± 0.0	0.1 ± 0.1	0.2 ± 0.7	0.1 ± 0.0	0.6 ± 0.7	0.1 ± 0.0	0.3 ± 0.1	0.6 ± 0.6	1.8 ± 2.4	0.3 ± 0.1	1.0 ± 0.8	1.3 ± 1.1	0.2 ± 0.1
						Freeze-thaw	1.3 ± 0.1	1.5 ± 0.1	1.3 ± 0.3	1.5 ± 0.2	0.8 ± 0.1	1.7 ± 0.3	1.2 ± 0.1	0.6 ± 0.0	0.6 ± 0.5	0.8 ± 0.1	1.1 ± 0.3	0.8 ± 0.1	1.1 ± 0.0	0.6 ± 0.2

Table S2. Primers used for real time quantitative PCR. The symbol ˇ denotes intron gap.

Gene	Locus	Left primer	Right Primer
HPL1	At4G15440	GCACGCCAAGˇATAAAAGGT	AAGATGCAACGTTGGAGAGG
HPL1	At4G15440	GGCCTAGCCTTGGGTTCTAC	AAGATGCAACGTTGGAGAGG
ACT8	At1G49240	GCTGGATTCGCTGGAGATGA	AGGTCTCCATCTCTTGCTCG
UBQE	At5G25760	CAGTCTGTGTGTAGAGCTATCATAGCAT	AGAAGATTCCˇCTGAGTCGCAGTT
LOX2	At3G45140	CTATGGAATCTTGCTAAGACTCATG	CGGCTGAACTTAGCTCTAATGCATA
AOS	At5G42650	CTTACCGGCGCATTGTTTAT	AAGTCAAAGCCGGTGAAATG
AOC1	At3G25760	AATACCGAAAACCCCAGACC	TTCGATCACAACGCAGAGAC
AOC2	At3G25770	GCCAAGAAGAACCTCACTGC	TCCGAGACCGAACATTAAGC
AOC3	At3G25780	GACCGAAAACTCCAGACCAA	TTCAGGAACGTGTTGGATCA
AOC4	At1G13280	TTCAGAACCATTTGCAGCAG	CGGTGATTCCTATGCGTTTT

Table S3. PCR markers used for mapping. SSLP, Simple Sequence Length Polymorphisms; SNP, Single-Nucleotide Polymorphism; TP-ARMS, Tetra-Primer Amplification Refractory Mutation System (Ye, Dhillon et al. 2001).

Marker name	Chromosome	Nearest locus	Genomic position	Marker type	Left primer(s)	Right primer(s)	PCR product length (bp)
**MN1.5**	1	AT1G03620	898430	SSLP	TTATTATCAAGATCAAAGATTGTATGGTTT	CTTGTTTTTATATCTGTTTGGTTTAATTGT	Col 308; C24 320
**CIW12**	1	AT1G27660	9621344	SSLP	AGGTTTTATTGCTTTTCACA	CTTTCAAAAGCACATCACA	Col 128; C24 100
**NGA280**	1	AT1G55840	20873698	SSLP	GGCTCCATAAAAAGTGCACC	CTGATCTCACGGACAATAGTGC	Col 105; C24 85
**ATPASE**	1	AT1G76030	28533837	SSLP	GTTCACAGAGAGACTCATAAACCA	CTGGGAACGGTTCGATTCGAGC	Col 85; C24 69
**NGA692**	1	AT1G76820	28836552	SSLP	AGCGTTTAGCTCAACCCTAGG	TTTAGAGAGAGAGAGCGCGG	Col 110; C24 90
**GOT10**	2	AT2G02540	693817	SSLP	GCGGTGAGTATCTCATTGCAT	AAGCCCATTCCCTAAACCAC	Col 214; C24 165
**GOT42**	2	AT2G21620	9249365	SSLP	ATTCATTTGTCGCATTTGCT	CAACAACACGAGCCACACTC	Col 207; C24 188
**GOT11**	2	AT2G41905	17493070	SSLP	GGTTTCCATGTTGGCTCAGT	GAACCAACGATTGGGCTAGA	Col 126; C24 147
**NGA172**	3	AT3G03340	786296	SSLP	CATCCGAATGCCATTGTTC	AGCTGCTTCCTTATAGCGTCC	Col 162; C24 150
**CIW11**	3	AT3G26610	9775545	SSLP	CCCCGAGTTGAGGTATT	GAAGAAATTCCTAAAGCATTC	Col 180; C24 230
**GOT47**	3	AT3G44735	16292750	SSLP	AACATGTTTTTGATAATCATCCATC	CATCTAAAAGATCTCCAGATTAAGTGA	Col 218; C24 250
**GOT48**	3	AT3G44755	16301990	SSLP	TCTCTTGATGGTGGTGTTGC	AGCCACGACCAGAAACTACG	Col 177; C24 189
**GOT39**	3	AT3G61550	22776727	SSLP	TCGTCGTTGGAGGTCTTGAT	ACGGGACAAGAACCATTGAG	Col 244; C24 229
**GOT45**	4	AT4G00880	367804	SSLP	ATCGAAAAACGAATCCATGC	AATATTCCCCACTTAGATATTCCA	Col 139; C24 154
**GOT46**	4	AT4G00890	372994	SSLP	GCCTCTGTTATTTAGGCTGAAAAT	CAGCTGTTGGTCAAACTGATACA	Col 224; C24 267
**MN4.2**	4	AT4G07410	4197601	SSLP	TAAGGTCAGACTATATGTTTACGTTTCATT	GTCATCCTCGTTTAAGTTACGATTG	Col 347; C24 357
**GOT3**	4	AT4G07840	4644916	SSLP	TCCTTAAAGCCCTTTGTTTATG	TTGTTGCTTTATTGGTTGGTTT	Col 175; C24 154
**GOT4**	4	AT4G09720	6131588	SSLP	GTGGATGCTAAGCGAATGGT	ATCGGGGAAGGGTGATGTAT	Col 209; C24 177
**GOT23**	4	At4G11750	7077901	SNP	CCATGTTTGGGTTTTGAGATC;<br>CCCATGTTAAGGTTTTGAGATT	TATTCACCAACGGTGCGTTC	Col, C24 206
**CIW6**	4	AT4G13575	7892624	SSLP	CTCGTAGTGCACTTTCATCA	CACATGGTTAGGGAAACAATA	Col 162; C24 155
**GOT22**	4	AT4G14400	8297901	SNP	GGTTATATCAGCGATGCCAAC;<br>GGTTATATCAGCGATAGCAAA	GCAAGGCCACATGTAAGGAT	Col, C24 187
**GOT31**	4	AT4G14590	8375401	SNP	ATCGAGCGAAGCGTTATGC;<br>CACATCGAACGAAGTGTAAAGC	TTCACATGAACCGTCTTCTCC	Col, C24 256
**GOT25**	4	AT4G14760	8484735	SNP	GAGTATTCAGGGACCAATAG;<br>GAGTATTCAGGGACCAATAT	TCTCCAAAAGAGCGCATTGT	Col, C24 243
**GOT33**	4	AT4G14965	8553090	SNP	TGAAGGTGGAGAGGTAGAAACTC;<br>TGAAGGTGGAGAGGTATGAACTT	CTTGGCAAGAGGTTCACAGT	Col; C24: 268
**GOT29**	4	AT4G15100	8625334	SNP	CGAACATATCTATCCGAGACTTG;<br>CGAGCATATCTATCTGAAACTCG	GGTGGTCGAATGATTCCAGA	Col, C24 322
**GOT32**	4	AT4G15290	8721450	SSLP	TGACAAAGGAAATATAGCATGTACG	AAGGGTTTGTTTGAGCATGG	Col 336; C24 302
**GOT35**	4	AT4G15396	8809580	SSLP	CCCAGATGGTGAGTATCCAA	TGTGATCCTAAGCGACTCATTT	Col 260; C24 278
**GOT30**	4	AT4G15440	8837250	SSLP	AAAATACAGAAGAAACGTGATACAAAA	GATTCGATTGTTCCCCAGAA	Col 282; C24 315

**GOT37**	4	AT4G15490	8852180	SSLP	ACGGTGTGTGCAATAACCAA	CCTCAGGCATCATCTCTCGT	Col 432; C24 369
**GOT6**	4	AT4G15545	8876513	SSLP	GCTGCAACTGACAACGAAAA	GCCGTGGCGTAGTAGTTTGT	Col 167; C24 179
**GOT27**	4	AT4G15890	9018101	SNP	TGGGAAGATTCTGGGAGGAG; GTGGGAAGATTTTAGGTGGAG	AAACTCTGATTGGCCCTCAA	Col, C24 310
**GOT9**	4	AT4G16444	9276263	SNP	TAAAGAAACGGCCCATTAAA; TGGGCTTCGTTGAAATTCTT	TCCTATTCATTCTAAATTTGGATACTT	Col 157; C24 173
**GOT17**	4	AT4G16770	9434101	SNP	GGTGGAAACCTGAGGCAGG; GGTGGAAACCTGAGGCAGC	CAGACCCAAACTGCGTTGTA	Col, C24 226
**GOT15**	4	AT4G17030	9584800	TP-ARMS	GAAGAAAATGTGAGTTTCTTCTTGTTCCT; CGAAAGACATAGTTTGTAGAATCGAAGC	CGTGAGTTTCTTCATACCCAAAGAAG; TTCTTTGGGAAGACTCTAAGAGAATGC	Col 104+166; C24 117+166
**GOT18**	4	AT4G17140	9630919	SNP	GCTGCTCTTTCTCGAGTCCA; GCTGCTCTTTCTCGAGTCCG	GCGGCGGTATTTATGCTTAG	Col, C24 180
**GOT19**	4	AT4g17565	9782371	SNP	TGGTCGATGAATTCCCTTATG	CATGACTTCAAGGTGTTATG; CATGACTTCAAGGTGTTATA	Col, C24 165
**GOT13**	4	AT4G18425	10180210	SNP	CTGGAGTCAAGTTCCCAGTTC	ATAATTGCAACAACCTAAAAAGA; ATAATTGCAACAACCTAAAACAA	Col, C24 159
**GOT16**	4	AT4G19185	10482401	TP-ARMS	GGTTATGCCTAACGTGTTTTCCGGAGTC; TACATCCGTAACGGTCCAAATCCACAGT	TACCGGTGTAACGGCGTCAGTAGCTC; CTGATTTACCCTACGCCGTCCGATTAAC	Col 199+365; C24 220+365
**GOT7**	4	AT4G20330	10977375	SSLP	TCTTGAGCAAGGTGCAAAAA	TAACTTCCAGGCGGCCTTAT	Col 144; C24 135
**GOT8**	4	AT4G33970	16272270	SSLP	TCGAGAGAGGTGAGAAGGTCA	CACATCTCTCATTGGATGATGAA	Col 138; C24 150
**T22D6-1ME**	5	AT5G08090	2584790	SSLP	ACGCTTCCCAAAAATCCACAA	CTCGGGAGACTCGCACGAT	Col 175; C24 230
**NGA139**	5	AT5G24620	8428133	SSLP	GGTTTCGTTTCACTATCCAGG	AGAGCTACCAGATCCGATGG	Col 174; C24 132
**GOT44**	5	AT5G24680	8453593	SSLP	TCACGCTTCCTCTCTCCCTA	GGTGATCGGTTTCGATTTGT	Col 197; C24 181
**GOT38**	5	AT5G38840	15551620	SSLP	CTCCGTGTCACCAATTTCAG	TGAAACCGAGAAATCGAAGG	Col 245; C24 230
**CER456385**	5	AT5G59160	23882280	SSLP	CACCGCCACCATACGAGCAT	TTCTGGCGGAGGATACTTCTTGAA	Col 210; C24 160

Ye, S., S. Dhillon, et al. (2001). "An efficient procedure for genotyping single nucleotide polymorphisms." Nucleic Acids Research **29**(17): e88.

Duan, H., M. Y. Huang, K. Palacio and M. A. Schuler (2005). "Variations in CYP74B2 (hydroperoxide lyase) gene expression differentially affect hexenal signaling in the Columbia and Landsberg erecta ecotypes of Arabidopsis." Plant Physiology **139**(3): 1529-1544.

Emanuelsson, O., H. Nielsen, S. Brunak and G. von Heijne (2000). "Predicting subcellular localization of proteins based on their N-terminal amino acid sequence." Journal of Molecular Biology **300**(4): 1005-1016.

Lamesch, P., T. Z. Berardini, D. Li, D. Swarbreck, C. Wilks, R. Sasidharan, R. Muller, K. Dreher, D. L. Alexander, M. Garcia-Hernandez, A. S. Karthikeyan, C. H. Lee, W. D. Nelson, L. Ploetz, S. Singh, A. Wensel and E. Huala (2012). "The Arabidopsis Information Resource (TAIR): improved gene annotation and new tools." Nucleic Acids Research **40**(Database issue): D1202-1210.
